# The Insulin-Like Growth Factor System and Neurological Complications in Diabetes

**DOI:** 10.1155/EDR.2003.235

**Published:** 2003

**Authors:** Anders A. F. Sima, Zhen-guo Li, Weixian Zhang

**Affiliations:** 1 Department of Pathology Wayne State University School of Medicine Room 9275, H. G. Scott Hall 540 East Canfield Avenue Detroit Michigan 48201 USA; 2 Department of Neurology Wayne State University School of Medicine Detroit Michigan USA; 3 Morris Hood Jr. Comprehensive Diabetes Center Wayne State University School of Medicine Detroit Michigan USA

## Abstract

The IGF system plays vital roles in neuronal development, metabolism, regeneration and survival. It consists of IGF-I, IGF-II, insulin, IGF-I-receptor, and those of IGF-II and insulin as well as IGF-binding proteins. In the last decades it has become clear that perturbations of the IGF system play important roles in the pathogenesis of diabetic neurological complications. In the peripheral nervous system IGF-I, insulin, and C-peptide particularly in type 1 diabetes participate in the development of axonal degenerative changes and contributes to impaired regenerative capacities. These abnormalities of the IGF system appear to be less pronounced in type 2 diabetes, which may in part account for the relatively milder neurological complications in this type of diabetes. The members of the IGF system also provide anti-apoptotic effects on both peripheral and central nervous system neurons. Furthermore, both insulin and C-peptide and probably IGF-I possess gene regulatory capacities on myelin constituents and axonal cytoskeletal proteins. Therefore, replenishment of various members of the IGF system provides a reasonable rational for prevention and treatment of diabetic neurological complications.


*Experimental Diab. Res.*, 4:235–256, 2003
Copyright © Taylor and Francis Inc.
ISSN: 1543-8600 print / 1543-8619 online
DOI: 10.1080/15438600390249691

Taylor & Francis
Taylor & Francis Group

### The Insulin-Like Growth Factor System and Neurological Complications in Diabetes

**Anders A. F. Sima,[1,2,3] Zhen-guo Li,[1,3] and Weixian Zhang[1,3]**

*Departments of [1]Pathology and [2]Neurology and [3]Morris Hood Jr. Comprehensive Diabetes Center, Wayne State University School of Medicine, Detroit, Michigan, USA*

**The IGF system plays vital roles in neuronal development, metabolism, regeneration and survival. It consists of IGF-I, IGF-II, insulin, IGF-I-receptor, and those of IGF-II and insulin as well as IGF-binding proteins. In the last decades it has become clear that perturbations of the IGF system play important roles in the pathogenesis of diabetic neurological complications. In the peripheral nervous system IGF-I, insulin, and C-peptide particularly in type 1 diabetes participate in the development of axonal degenerative changes and contributes to impaired regenerative capacities. These abnormalities of the IGF system appear to be less pronounced in type 2 diabetes, which may in part account for the relatively milder neurological complications in this type of diabetes. The members of the IGF system also provide anti-apoptotic effects on both peripheral and central nervous system neurons. Furthermore, both insulin and C-peptide and probably IGF-I possess gene regulatory capacities on myelin constituents and axonal cytoskeletal proteins. Therefore, replenishment of various members of the IGF system provides a reasonable rational for prevention and treatment of diabetic neurological complications.**

**Keywords** C-Peptide; Diabetic Encephalopathy; Diabetic Neuropathy; IGF's; Insulin

Received 23 December 2002; accepted 6 April 2003.

This study was supported by grants from JDRF (AAFS), The Thomas Foundation (AAFS), and the Morris Hood Jr. Diabetes Center (ZGL).

Address correspondence to Anders A. F. Sima, MD, PhD, Department of Pathology, Wayne State University, Room 9275, H. G. Scott Hall, 540 East Canfield Avenue, Detroit, MI 48201, USA. E-mail: asima@med.wayne.edu

### INTRODUCTION

### Definition of the Insulin-Like Growth Factor (IGF) System

The IGF system consists of IGF-I, IGF-II, insulin, insulin-like growth factor receptors (IGF-IRs), insulin receptor (IR), and IGF-binding proteins (IGFBPs) (Le Roith, 1999). IGF-I and IGF-II are single-chain polypeptides similar to that of proinsulin and their amino acid sequences share extensive homologies with insulin (Blundell et al., 1983; Rinderknecht and Humbel, 1978). Both IGF-I and IGF-II signal predominantly via the IGF-IR, whereas insulin acts primararily through the IR. IGF-IR and IR are tyrosine kinase receptors characterized by $\alpha_2\beta_2$ heterotetramers that are held by disulfide bonds (Ullrich and Schlessinger, 1990). The $\beta$ subunit of IGF-IR shares a high homology (84%) with that of IR, whereas the cysteine-rich pockets of the $\alpha$ subunit of IGF-IR shows low homology (48%) with that of IR (Ullrich et al., 1986). IGF-I and insulin bind weakly to each other's receptors, with a 1000-fold lower affinity than that for the cognate receptor (Kjeldsen et al., 1991). IGF-IIR is a receptor that differs from IGF-IR and IR, as it has a single transmembrane protein bearing binding sites for mannose-6-phosphate–containing residues (Kiess et al., 1994), while IGF-IR and IR are tyrosine kinase receptors (Figure 1).

In spite of their structural similarities, IGF-IR and IR mediate different effects: mainly metabolic effects for IR and predominantly growth effects for IGF-IR. The mechanisms underlying the specificities of the IGF-IR and IR signaling are not totally clear. Several mechanisms are probably of importance, such as differences in tissue distribution, modulation of ligand binding by local environments, such as IGFBPs, and differences in

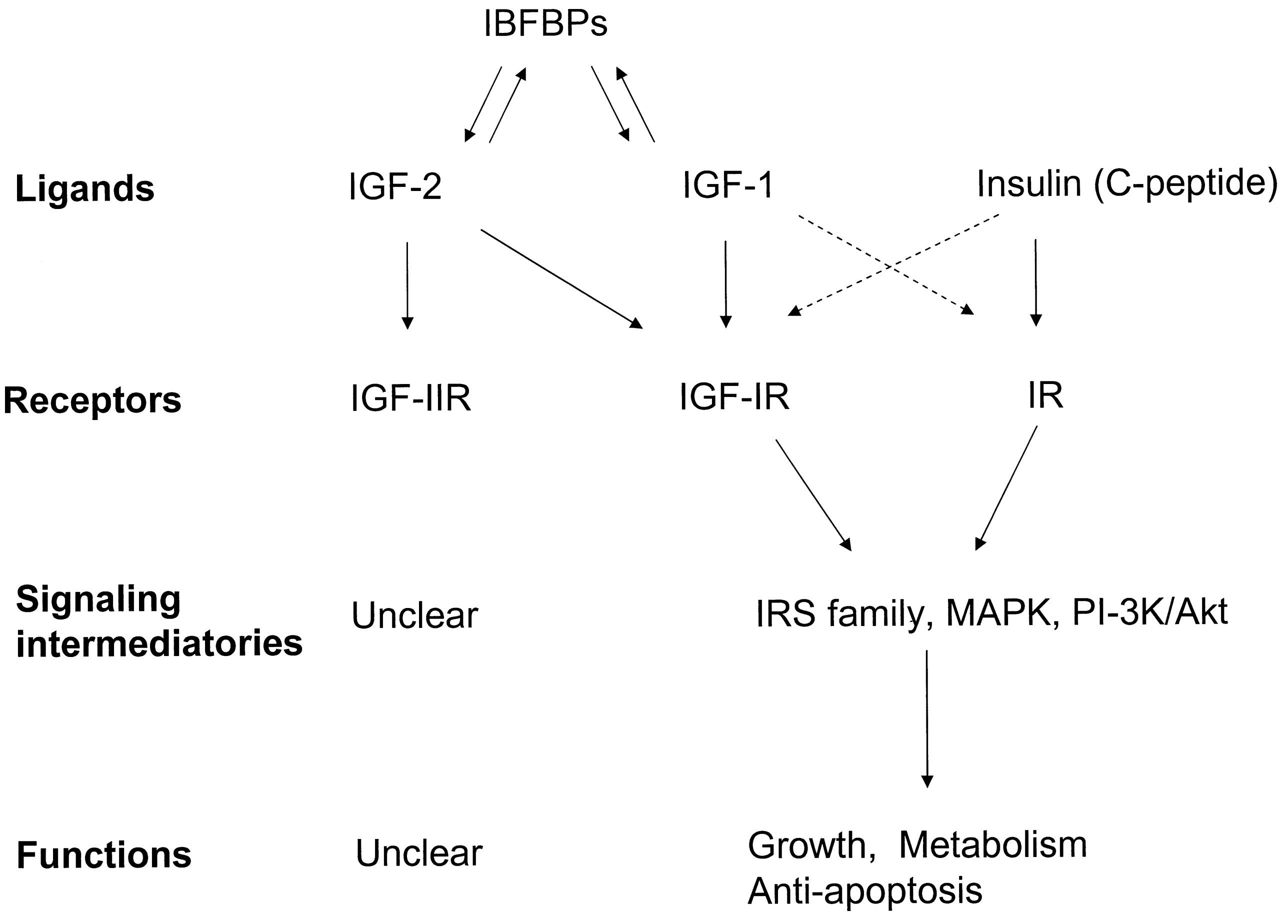

**FIGURE 1**

Summary of IGF system. The IGF system consists of IGF-I, IGF-II, insulin, insulin-like growth factor receptors (IGF-IR and IGF-IIR), insulin receptor (IR), and IGF-binding proteins (IGFBPs). In addition to these are the insulinomimetic C-peptide. IGF-I and IGF-II are single-chain polypeptides and bind to IGF-IR with high affinity, whereas insulin acts primarily through the IR. IGF-IIR is a single transmembrane peptide bearing binding sites for mannose-6-phosphate, its signaling and function being unclear. IGF-I and IGF-II are interactive with IGFBPs, whereas insulin does not bind to IGFBPs. Although IGF-IR and IR utilize similar signaling pathways, IGF-IR and IR mediate different effects: mainly metabolic effects for IR and predominantly growth effects for IGF-IR. In addition, recent studies have shown that both IGF-IR and IR mediate antiapoptotic effects. The weak binding of IGF-I/IGF-II to IR and insulin to IGF-IR are indicated with dotted lines. C-peptide shows insulin-like effects, although its receptor binding is not clear. It appears to regulate the expression of both IGF-I, IGF-II, IGF-IR, and IR possibly, via NF-$\kappa$B.

downstream substrates (Blakesley et al., 1996). To define functional specificity, mutations of the various domains of IGF-IR and IR have been performed. For IGF-IR, the tyrosine cluster 1131, 1135, and 1136 (Gronborg et al., 1993; Li et al., 1994) and tyrosine 950 (Miura et al., 1995a) are crucial for both mitogenic and transformation activities, but not for antiapoptotic activity. Substitution of tyrosine 1251 with phenylalanine results in a loss of the antiapoptotic activity (O'Connor et al., 1997) and a reduced transformation activity, but it does not affect mitogenicity (Miura et al., 1995b). These data indicate that domains required for inhibition of apoptosis are distinct from those essential for both transformation and mitogenicity. With respect to the IR, mutation experiments have established relationships between phosphorylation of specific receptor sites and activation of the receptor kinase activity (Avruch et al., 1990; Flores-Riveros et al., 1989; Tavare and Denton, 1988; Tornqvist et al., 1987; White et al., 1988). The Tyr-1150 domain, including three tyrosine residues at positions 1146, 1150, and 1151, is crucial for IR tyrosine kinase activity (Hashimoto et al., 1992). Mutation at the ATP binding sites (Lys-1018 or Lys-1030 in the $\beta$ subunit of IR) leads to loss of insulin-stimulated kinase activity (Chou et al., 1987; Ebina et al., 1987). In contrast, the phosphorylation of tyrosines 1316 and 1322 in the carboxyl-terminal region do not influence the receptor kinase activity (Maegawa et al., 1988; Myers et al., 1991; Takata et al., 1991). These results suggest that specifically located tyrosine domains of the IR and IGF-IR may be required for specific ligand-induced cellular functions.

The IGFs are associated with high-molecular-weight carrier proteins, IGFBPs, present in the circulation and brain (Baxter, 2000). Currently, six different IGFBPs have been identified. They share a cysteine-rich region but differ substantially in other regions (Baxter, 1994; Katz et al., 1995). The interaction of IGFs with their receptor is controlled by IGFBPs. Most of the IGFs in the circulation are bound to IGFBPs, because the affinity of IGFs for the IGFBPs is greater than that for the IGF receptors. When the affinity of IGFBPs for IGFs is reduced, the IGFs are released to interact with the IGF receptors (Blakesley et al., 1996). Therefore, IGFBPs act as partitioning agents controlling the amount of IGF that is available for receptor association. Three factors have been found to be important for regulation of IGFBP affinity: (a) association with extracellular matrix proteins reduces IGFBP affinity (Jones et al., 1993); (b) proteolytic cleavage of IGFBP reduces greatly the affinity for IGFs (Clemmons et al., 1998); and (c) phosphorylation of serine residues on IGFBP-1 results in 6- to 7-fold enhancement of its affinity for IGF-I (Jones et al., 1991). In the rat, IGFBP-3 is the principal carrier protein for IGFs in the circulation (Katz et al., 1995). Under physiological conditions, IGFBP-2, -3, -4, -5, and -6 are expressed in the central nervous system (CNS), IGFBP-2 being the predominant IGFBP (Walter et al., 1999). Diabetes affects the levels of IGFBPs. IGFBP-1 is increased in streptozotocin (STZ)-induced diabetic rats and patients (De La Puente et al., 2000; Landau et al., 1995; Luo and Murphy, 1991; Park et al., 1998; Price et al., 1997; Rodgers et al., 1995), whereas IGFBP-3 is decreased (Cinaz et al., 1996; Graubert et al., 1991). The expression of IGFBP genes is regulated by insulin: insulin inhibits IGFBP-1 and stimulates IGFBP-3 gene transcription (Graubert et al., 1991; Katz et al., 1998).

In summary, the IGF system is a complicated system consisting of ligands, receptors, and interacting binding proteins. The components of this system interact with each other in a complicated mode. The ligands of the IGF system may interact with other receptors besides their own, for example, IGF-I binds to the IR and insulin binds to IGF-IR, however, with low affinities. Therefore, IGF-I signaling is not an isolated phenomenon. It is modulated by the presence of IGFBPs, and probably by insulin and maybe by C-peptide. These interactive relationships account for the intricate function of the IGF system. As mentioned earlier, the IGF system consists of IGF-I, IGF-II, IGF-IR, IGF-IIR, IGFBPs, and IR. To this series of associated trophic factors, we propose to add the proinsulin C-peptide as an interactive member of the system for reasons that will become clear.

### Function of the IGF System

The IGF system plays a vital role in mediating growth, development, metabolism, and survival of many tissues and organs. In neuronal tissues, the IGF system supports the survival of neurons (Ang et al., 1992; Zackenfels et al., 1995; Zackenfels and Rohrer, 1993; Zheng et al., 2002) and Schwann cells (Cheng and Feldman, 1997; Syroid et al., 1999), regulates neuronal differentiation (Arsenijevic and Weiss, 1998; Brooker et al., 2000; Feldman et al., 1997; Morrione et al., 2000; Ochoa et al., 1997), enhances regeneration in cultured adult rat sensory neurons (Fernyhough et al., 1993), stimulates neurite outgrowth (Kim et al., 1997; Rind and von Bartheld, 2002; Wang et al., 1992; Zhang et al., 2001), enhances oligodendrocyte development (D'Ercole et al., 1996; Dubois-Dalcq and Murray, 2000; Jiang et al., 2001; McMorris and McKinnon, 1996), promotes myelination of the nervous system (Cheng et al., 1999; Copelman et al., 2000; Hetts et al., 1997; Mozell and McMorris, 1991; Russell et al., 2000; Ye et al., 1995; Zackenfels et al., 1995), rescues neuronal loss following cerebral hypoxic-ischemic injury (Gluckman et al., 1992; Guan et al., 1993; Williams et al., 1995), and protects against neuronal apoptosis (Zawada et al., 1998; Zhang et al., 2002). IGF-I has been used in the treatment of several disorders, including growth deficiency, osteoporosis, catabolic disorders, diabetes, and neurodegenerative disorders (Dore et al., 1997). The rather unique propensity of IGF-I to act on a variety of neuronal cells might provide a general means of reducing or slowing down neuronal losses that occur following various brain insults.

IGF-I regulates calcium ion channel currents and neuronal excitability (Blair and Marshall, 1997; Hall et al., 1995; Ristic et al., 1998; Selinfreund and Blair, 1994). The rapid action of IGF-I on the voltage sensitivity of L-type calcium channel indicates that IGF-I, in addition to function as a mitogen with long-term effects, also acts as a rapid neuromodulator (Bence-Hanulec et al., 2000). IGF-I rapidly promotes phosphorylation of the $\alpha 1$ subunit of the calcium L-channel (Bence-Hanulec et al., 2000) and activates the phosphatidylinositol (PI) 3-kinase/Akt pathway (Blair et al., 1999), which may promote the survival of cerebellar granule neurons. Besides calcium channels, IGF-I up-regulates potassium channels through PI 3-kinase, PDK1, and SGK1 pathways to exert the proliferative action of the growth factor (Gamper et al., 2002) and it may modulate sodium channel expression (Craner et al., 2002).

Recent studies show that the IGF system plays antiapoptotic effects in various types of cells, including hematopoietic cells (Kelley et al., 1998; Zumkeller, 2002), osteoblasts (Hill et al., 1997; Kawakami et al., 1998; Tumber et al., 2000), fibroblasts (Buckley et al., 2002; Valentinis et al., 1999), melanoma cells (Kanter-Lewensohn et al., 2000), myoblasts (Foulstone et al., 2001; Hong et al., 2001), mouse embryonic fibroblast NIH3T3

cells (Sell et al., 1995), NWTb3 cells, NIH3T3 cells expressing the normal human IGF-IR (Heron-Milhavet et al., 2001), and epithelial cells (Moorehead et al., 2001; Wilkins et al., 2002). In neuronal cells, it promotes neuronal survival following ultraviolet (UV) irradiation (Kulik et al., 1997) and serum deprivation in PC12 cells (Parrizas et al., 1997b). It inhibits apoptosis of cerebellar granule neurons induced by low potassium (D'Mello et al., 1997; Galli et al., 1995), and protects against apoptosis of doral root ganglia (DRG) and Schwann cells exposed to high glucose (Delaney et al., 2001; Russell et al., 1999; Vincent et al., 2002) and neuroblastoma SH-SY5Y cells exposed to high concentration of mannitol or glucose (Cheng and Feldman, 1998; Matthews and Feldman, 1996; Singleton et al., 1996; van Golen and Feldman, 2000; Vestling et al., 2001; Vincent et al., 2002; Zhang et al., 2001) or to 3-morpholinosydnonimine (SIN-1), a peroxynitrite donor (Saeki et al., 2002). On the other hand, decreased IGF-I gene expression is found in Purkinje cells undergoing apoptosis (Zhang et al., 1997) and inhibition of IGF-I activity appears to contribute to premature apoptosis of cerebellar granule neurons in the weaver mutant mice (Zhong et al., 2002). IGF-I and IGF-II (3 to 100 ng/mL) each prevents glucose deprivation–induced neuronal damage in a dose-dependent manner in rat hippocampal and septal neuronal cell cultures, whereas insulin exhibits a protective effect at high concentrations (greater than 1 $\mu$g/mL) and epithelial growth factor (EGF) does not show any protective effect at all (Cheng and Mattson, 1992). This latter study is in accordance with the results of other investigators, who show that the IGF-I, but not EGF and fibroblast growth factor (FGF), protects against c-Myc–induced apoptosis in Rat-1 fibroblasts via the activation of IGF-IR (Harrington et al., 1994), and that the IGF-I, but not acidic FGF (aFGF), basic FGF (bFGF), nerve growth factor (NGF), and platelet-derived growth factor (PDGF), rescues neuroblastoma SH-SY5Y apoptosis induced by hyperosmolarity (van Golen and Feldman, 2000). Therefore, it is well documented that IGF-I exerts antiapoptotic effects via IGF-IR signaling.

The IGF-IR signaling may utilize multiple pathways in a cell type–specific manner to exert its antiapoptotic activity: the PI 3-kinase/Akt pathway in Rat-1 cells, promyeloid cells, PC 12 cells, and DRG neurons (Dudek et al., 1997; Liu et al., 1997; Parrizas et al., 1997b); the MAP (mitogen-activated protein) kinase pathway in PC 12 cells (Cano et al., 1996); the Crk-II–enhanced Ras-dependent but Raf-1/MAP kinase–independent pathway in human embryonic kidney 293 cells (Parrizas et al., 1997a); the stress-activated p38 MAP kinase and c-Jun N-terminal kinase (JNK) pathway in neuroblastoma cells (Cheng and Feldman, 1998; Okubo et al., 1998; Zhang et al., 2001); p38 MAP kinase pathway in NWTb3 cells (Heron-Milhavet et al., 2001); up-regulation of Bcl-xl and activation of PI 3-kinase in Baf-3 cells (Leverrier et al., 1999); and PI 3-kinase in mouse neocortical neuronal cell cultures (Ryu et al., 1999). Among the multiple signaling pathways that are involved in the IGF-IR–mediated antiapoptotic effect, PI 3-kinase/Akt pathway plays a key role and MAP kinase pathways are implicated. As illustrated in Figure 2*a*, binding of IGF-I/IGF-II to IGF-IR activates autophosphorylation of the receptor and phosphorylation of several intracellular substrate proteins (IRS family and Shc), and leads to activation of PI 3-kinase/Akt pathway and MAP kinase pathway (Figure 2). The activated Akt inactivates proapoptosis factors, such as Bad, p53, and caspase-3 (Bai et al., 1999; Brunet et al., 1999; Kermer et al., 2000; Yamaguchi et al., 2001); increases antiapoptosis factors, such as Bcl2 and Bclx (Leverrier et al., 1999; Li et al., 2001; Zhang et al., 2002); activates transcriptional factors, such as nuclear factor [NF]-$\kappa$B (Heck et al., 1999; Li et al., 2001) and cyclic AMP response element–binding protein (CREB) (Pugazhenthi et al., 1999). It decreases forkhead transcription factor (FKH) (Brownawell et al., 2001; Kops and Burgering, 2000), which reduces expression of FKH target genes, including Fas ligand, and therefore decreases Fas-mediated apoptosis (Vincent and Feldman, 2002). With respect to the MAP kinase pathway, the activated extracellular signal–regulated kinase (ERK) inactivates Bad (Bonni et al., 1999; Scheid et al., 1999) and the p38 MAP kinase activates CREB (Bonni et al., 1999; Pugazhenthi et al., 1999). Overall activation of these pathways results in antiapoptotic effects.

In contrast to IGF-IR, the IR-mitigated antiapoptotic effects are not totally clear. As shown in Figure 2*b*, insulin inhibits serum withdrawal induced Chinese hamster ovarian (CHO) cell apoptosis via a PI 3-kinase–dependent pathway (Lee-Kwon et al., 1998) and a Raf-1–dependent pathway, which leads to activation of NF-$\kappa$B (Bertrand et al., 1998) and the NF-$\kappa$B–dependent survival genes encoding tumor necrosis factor receptor–associated factor 2 (TRAF2) and manganese superoxide dismutase (Mn-SOD) (Bertrand et al., 1999). Insulin rescues serum-deprived immortalized brown adipocytes from apoptosis through PI 3-kinase/Akt pathways (Navarro et al., 2000), prevents cardiomyocytes from oxidative stress–induced apoptosis through activation of PI 3-kinase/Akt (Aikawa et al., 2000), and inhibits apoptosis of macrophage THP-1 cells, via a PI 3-kinase–dependent pathway (Iida et al., 2002). Data from our laboratory show that insulin activates p38 kinase, inhibits JNK, and promotes nuclear translocation of NF-$\kappa$B in neuroblastoma SH-SY5Y cells, indicating that these pathways are involved in IR-mediated antiapoptotic effects (Zhang et al., 2002).

Furthermore, recent studies show that other components of the IGF system besides IGF-IR, including IR and IGFBPs, play antiapoptotic roles. Insulin exerts an antiapoptotic effect in various cell types, including external granular layer neurons in rat cerebellar slice cultures (Tanaka et al., 1995), Rat-1 fibroblasts

A.

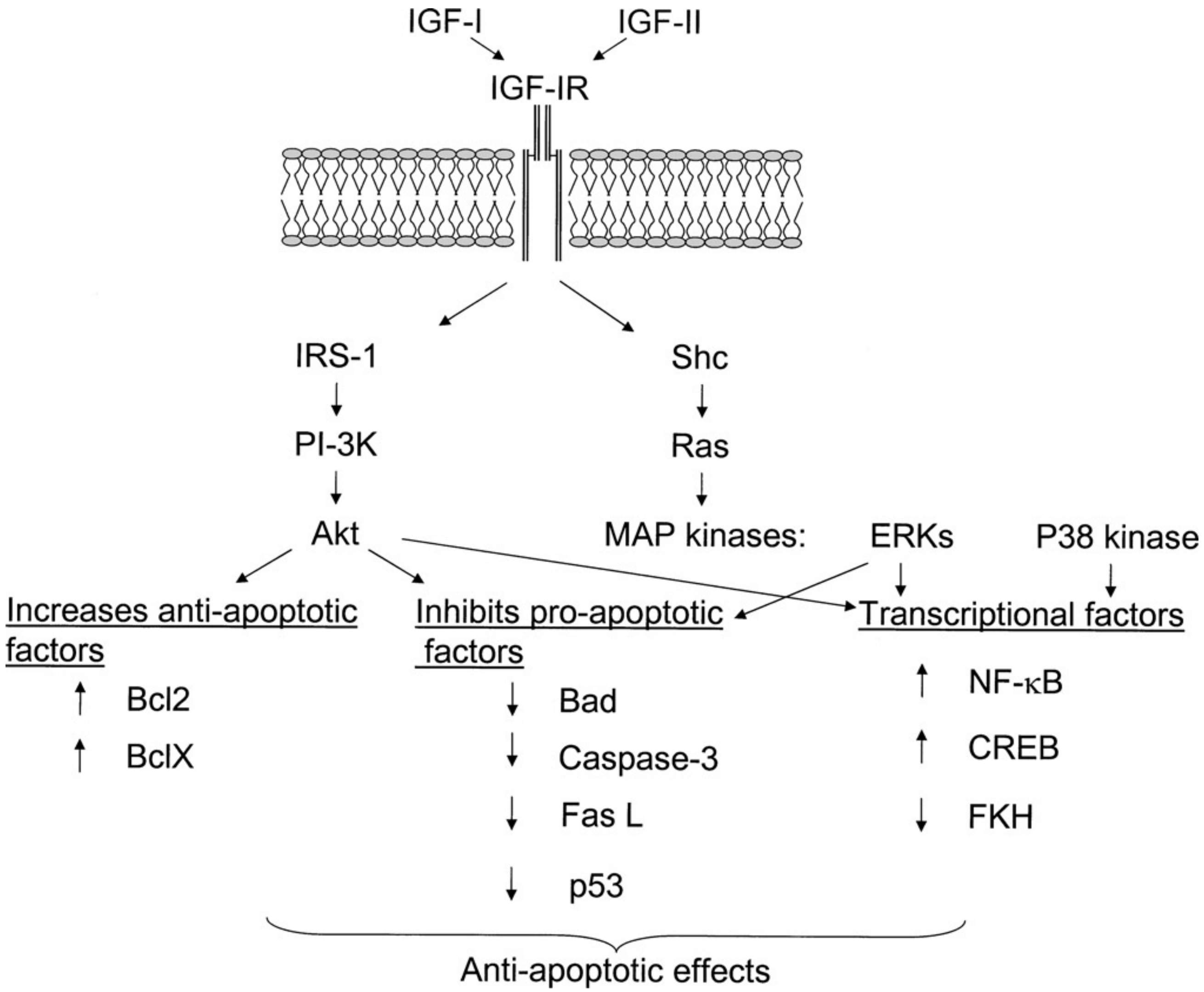

**FIGURE 2**
Summary of IGF-IR and IR pathways mediating antiapoptotic effects by IGFs. (*A*) IGF-IR signaling pathways. Binding of IGF-I/IGF-II to IGF-IR activates autophosphorylation of the receptor and of several intracellular substrate proteins (IRS family and Shc), leading to activation of PI 3-kinase/Akt pathway and MAP kinase pathway. The activated Akt inactivates proapoptosis factors, such as Bad, p53, and caspase-3); increases antiapoptosis factors, such as Bcl2 and Bclx); and activates transcriptional factors, such as nuclear factor (NF)-$\kappa$B and cyclic AMP response element binding protein (CREB). It decreases forkhead transcription factor (FKH), which reduces expression of FKH target genes, including Fas ligand and therefore decreases Fas-mediated apoptosis. With respect to the MAP kinase pathway, the activated extracellular signal–regulated kinase (ERK) inactivates Bad. The p38 MAP kinase activates CREB. Overall activation of these pathways results in antiapoptotic effects. (*B*) IR pathways. The IR-mitigated antiapoptotic effects are not totally clear. Insulin inhibits apoptosis via a PI 3-kinase/Akt pathway and a Raf-1–dependent pathway, each of wich leads to activation of NF-$\kappa$B. The NF-$\kappa$B–dependent survival genes encoding tumor necrosis factor receptor–associated factor 2 (TRAF2) and manganese superoxide dismutase (Mn-SOD). Data from our laboratory show that insulin activates p38 kinase, inhibits JNK, and promotes nuclear translocation of NF-$\kappa$B in neuroblastoma SH-SY5Y cells (Zhang et al., 2002). All these pathways are involved in IR-mediated antiapoptotic effects.
*(Continued)*

B.

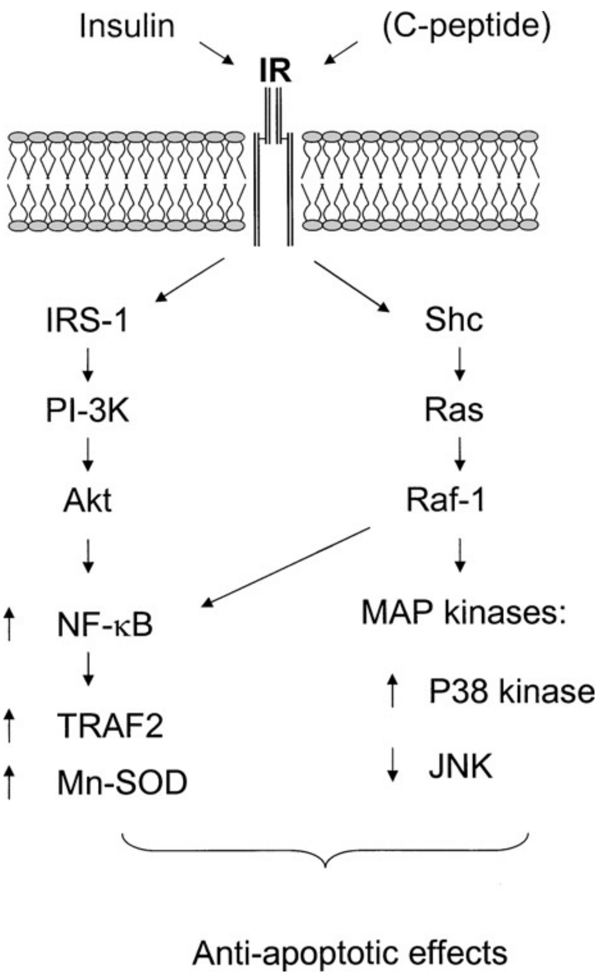

**FIGURE 2**
(Continued)

(Kummer et al., 1997), macrophages (Iida et al., 2002), serum-deprived immortalized brown adipocytes (Navarro et al., 2000), cardiomyocytes of neonatal rats (Aikawa et al., 2000), and cultured rat adipocytes (Qian et al., 2001). In many studies, this effect was observed at high concentrations of insulin. It was therefore believed that the observed antiapoptotic function was mediated via IGF-IR and not by IR itself. However, Lee-Kwong et al. (1998), Bertrand et al. (1998), and Kummer et al. (1997) independently reported that low doses of insulin rescue CHO cells CHO or Rat-1 fibroblasts from apoptosis induced by serum starvation. These results indicate that IR indeed mediates an antiapoptotic effect. We have shown that low doses of insulin stimulate neurite outgrowth, enhance cell proliferation, and protect against high glucose–induced apoptosis of neuroblastoma SH-SY5Y cells (Zhang et al., 2001, 2002). These data support the notion that insulin plays an antiapoptotic function via its own receptors. Furthermore, the combination of low doses of C-peptide and insulin give rise to an additive effect on neurite outgrowth, cell proliferation, and antiapoptosis in SH-SY5Y cells (Zhang et al., 2001, 2002), indicating that the proinsulin C-peptide may play an active role in the interactions of the IGF system.

IGFs are implicated in neurodegenerative disorders. Serum levels of insulin and IGFs and their binding proteins are perturbed in various human neurodegenerative disorders, such as Alzheimer's disease, amyotrophic lateral sclerosis, cerebellar ataxia, ataxia-telangiectasia (AT), and Charcot-Marie-Tooth 1A (CMT-1A) disease (Busiguina et al., 2000). Similarly, serum insulin and IGF-I levels are decreased in diabetic patients (Bereket et al., 1996; Cinaz et al., 1996; Clauson et al., 1998; Fujihara et al., 1996; Normann et al., 1994; Rieu and Binoux, 1985; Tan and Baxter, 1986). Furthermore, neuronal apoptosis has been demonstrated in both neurodegenerative disorders (Mattson, 2000; Mizuno et al., 1998; Tatton et al., 1997) and in experimental type 1 diabetes (Li et al., 2002a). These results suggest that similar mechanisms may be operable in a variety of neurodegenerative disorder and CNS complications of diabetes. However, more work is required to elucidate the potential pathogenetic roles of IGFs in neurodegenerative disorders, such as Alzheimer's disease and the CNS complication of diabetes.

IGFBPs also play a role in the regulation of apoptosis. IGFBP-5 decreases apoptosis in human breast cancer cells (Perks et al., 2000), whereas IGFBP-3 increases apoptosis in the same cells (Baxter, 2001; Perks et al., 2000). Another antiapoptotic factor that may be related to the IGF system is the IGF-I–cleaving protease. It releases from the amino-terminal of IGF-I molecule a tripeptide Gly-Pro-Glu (Guan et al., 1999), which protects neuronal death in Huntington's disease, Parkinson's disease, cerebral hypoxic-ischemic injury, and *N*-methyl-D-aspartate (NMDA) toxicity (Alexi et al., 1999; Guan et al., 2000; Sara et al., 1989; Sizonenko et al., 2001).

### NEUROLOGICAL COMPLICATIONS OF DIABETES

Diabetes is a chronic metabolic disorder that is reaching pandemic proportions. The two major forms are type 1 (or insulin-dependent) and type 2 (non–insulin-dependent) diabetes mellitus. They both have in common hyperglycemia and are apt to develop chronic complications such as nephropathy, neuropathy, retinopathy, and encephalopathy and to predispose to cardiovascular disease. Although both types of diabetes do develop chronic complications, they differ in their expression and progression, which is likely a reflection of differences in underlying pathogenetic mechanisms. For a long time, it was believed, and unfortunately still is to some extent, that hyperglycemia was the only culprit underlying the chronic complications of diabetes and hence by definition they were the same in the two types of diabetes. In recent years, however, it has become increasingly evident that factors such as insulin deficiency and associated C-peptide deficiency, as well as progressive perturbations of trophic factors in type 1 diabetes play important roles in the pathophysiology in most of the chronic complications and may be responsible for the usually more severe forms of complications in this type of diabetes (Kohner, 1987; Marshall and Alberti, 1989; Mogensen, 1998; Sima et al., 1988; Sugimoto et al., 2000a).

The IGF system has been implicated in the pathogenesis of the chronic complications, such as the role of IGF-I in the development of diabetic neuropathy (Ishii, 1995), cardiovascular disease in type 2 diabetes (Janssen and Lamberts, 2002), and in diabetic angiopathy (Chiarelli et al., 2000; Janssen and Lamberts, 2000). In this article, we will focus on the neurological complications and IGFs, their neurotrophic effects that support nerve integrity and regeneration, and their protective effects on neuronal survival (Zhuang et al., 1997).

### PERIPHERAL NEUROPATHY

Diabetic neuropathies as a group are the most common chronic complication of diabetes mellitus (Greene et al., 1997), but remain probably the least understood complication. The prevalence of diabetic neuropathy varies from 10% within 1 year of diagnosis of diabetes to 50% in patients with diabetes for more than 25 years (Dyck et al., 1993; Pirart, 1977; Sima, 1997; Vinik et al., 1992). Diabetic neuropathies include several distinct syndromes, among which distal symmetric polyneuropathy, often associated with diabetic autonomic neuropathy, is the most common and is referred to as diabetic polyneuropathy (DPN). The different syndromes affecting peripheral nerve can be separated into rapidly reversible manifestations and chronic progressive syndromes. The latter can be divided into symmetric polyneuropathies and focal/multifocal neuropathies (Sima, 1997; Sima et al., 1997c; Thomas, 1997).

The mechanisms underlying DPN are multiple and appear to involve several interrelated metabolic abnormalities consequent to hyperglycemia and insulin and C-peptide deficiencies (Greene et al., 1992, 1997; Sima, 1996; Sima and Sugimoto, 1999; Sima et al., 2001b; Sugimoto et al., 2000a, 2000c).

The results from the Diabetes Control and Complications Trial (DCCT) support this notion. Intensive glycemic control for 5 years reduced the incidence of clinical neuropathy by 60% in type 1 patients (The DCCT Research Group, 1993). The fact that strict hyperglycemic control did not completely prevent diabetic neuropathy (or any of the other complications), suggests additional pathogenetic attributes such as genetic predispositions, insulin/C-peptide deficiencies, and perturbations of trophic factors.

DPN shows a more severe clinical course in type 1 patients, associated with more severe structural changes compared to duration-matched type 2 patients (Dyck et al., 1999; Sima et al., 1988). Perturbations in the expression of insulin receptor and that of other growth factors and their receptors, either does not occur or are mild in a type 2 animal model (Pierson et al., 2002a). We and others (Sima et al., 2000, 2001a; Yagihashi, 1995; Yagihashi et al., 1994) have explored some of the differences in DPN in type 1 versus type 2 animal models. Comparisons of two isohyperglycemic models have demonstrated functional, biochemical, molecular, and structural differences, which have been ascribed to the presence/absence of insulin and/or the insulinomimetic C-peptide (Sima et al., 2000, 2001b). Type 1 DPN is in both experimental models and patients characterized by severe axonal atrophy resulting in a dying-back–type axonopathy with progressive nerve fiber loss. This latter phenomenon is probably in part due to impaired nerve fiber regeneration. Furthermore, progressive nodal and paranodal changes occur in type 1 but not in type 2 DPN. Type 2 DPN, on the other hand, shows a milder axonal atrophy, mild axonal loss, and normal regenerative capacity, but an increased frequency of segmental demyelination (Sima et al., 1986, 1988, 1997c; Sima and Sugimoto, 1999; Sugimoto et al., 2000a; Yagihashi, 1995; Yagihashi et al., 1994). It was recently shown that insulin treatment enhances the expression of IGF-I in sural nerves of diabetic patients (Grandis et al., 2001) and that C-peptide corrects partially the decreased activity of IGFs in hippocampus of type 1 diabetes (Li et al., 2002b), supporting the above notion.

### IGFs and DPN

Unlike NGF, IGFs exert their neurotrophic effects on most peripheral nerve fibers, be they sensory, motor, or autonomic. The neurotrophic support by the IGF system was recognized in the mid 1980s (Ishii et al., 1985; Recio-Pinto and Ishii, 1984) when the effects of IGF-I and IGF-II on neurite outgrowth and cell survival were described. Similar effects by insulin and C-peptide were only described recently (Li et al., 2001, 2002b; Pierson et al., 2002a, 2002b, 2003). Like peripheral nerve expression of NGF, the deficit in IGF-I expression occurs gradually and is established at 6-week duration in the type 1 diabetic BB/Wor-rat (Pierson et al., 2002a; Tomlinson and Fernyhough, 2000; Xu and Sima, 2001).

### *Clinical Studies*

Circulating IGF-I activity is markedly reduced in both type 1 and type 2 diabetes (Arner et al., 1989; Ekman et al., 2000) and does not appear to correlate with the degree of glycemic control (Ekman et al., 2000). Crosby et al. (1992) showed decreased IGF-I and elevated IGFBP-I levels in type 1 patients with peripheral neuropathy. In type 2 patients, those with clinical DPN showed significantly lower levels of IGF-I compared to diabetic patients without DPN or nondiabetic control subjects (Guo et al., 1999). Similar results were reported by Migdalis et al. (1995), who demonstrated decreased IGF-I plasma levels as well as decreased densities of IGF-IR on red blood cells in type 2 patients with sensory and autonomic neuropathy, compared to non-neuropathic patients and nondiabetic control subject. These findings suggest an association between DPN and impaired IGF-I activity. Of the other IGFs, insulin and C-peptide are certainly deficient in type 1 diabetes, whereas they may be increased systemically, at least in early insulin resistant type 2 diabetes. As to whether the peripheral insulin resistance of type 2 diabetes also applies to the peripheral and/or central nervous system is not known. These differences in the availability of IGFs, including insulin and C-peptide, are likely to account for some of the neurotrophic deprivations seen in type 1 diabetes and may relate to the generally more severe manifestations of DPN in type 1 diabetes (Sima and Sugimoto, 1999; Sugimoto et al., 2000a).

*Experimental Studies*

Neurotrophic factor activities in peripheral nerve tissues have been studied in both type 1 and type 2 diabetic animal modes. In both the STZ and BB/Wor rats, there is a progressive reduction in NGF mRNA and other neurotrophins with duration of diabetes (Fernyhough et al., 1994, 1998; Pierson et al., 2002a, 2002b; Riaz and Tomlinson, 1996; Tomlinson and Fernyhough, 2000; Xu and Sima, 2001). Circulating levels of serum IGF-I are diminished (Bornfeldt et al., 1989; Sima et al., 1997b). In contrast, in type 2 animal models such as the ZDF(fa/fa) rat and the BB/Z rat (Sima et al., 1997a), IGF-I expression is not reduced in peripheral nerve or spinal cord (Pierson et al., 2002a, 2002b, 2003; Wuarin et al., 1994, 1996; Zhuang et al., 1997), whereas IGF-II gene expression is reduced in peripheral nerve of the ZDF(fa/fa) rat. Likewise, the expression of IGF-I and its receptor is significantly down-regulated in DRG and the superior cervical ganglion in type 1 animal models (Bitar et al., 1997; Craner et al., 2002; Ishii and Lupien, 1995; Pierson et al., 2003; Wuarin et al., 1994; Xu et al., 2002), but not in the type 2 BB/Z rat (Pierson et al., 2003). Because IGFs regulate the gene expression of neuroskeletal proteins such as tubulin and neurofilaments, which in turn are determinants for axonal caliber, these discrepancies between their expression in type 1 and type 2 animal models may account for the substantially milder axonal atrophy seen in the latter (Murakawa et al., 2002; Sima et al., 2000; Yagihashi et al., 1994) (Figure 3). This notion is supported by experiments demonstrating that infusion of either IGF-I or IGF-II into crushed sciatic nerves increases the distance of motor and sensory axon degeneration (Glazner et al.,

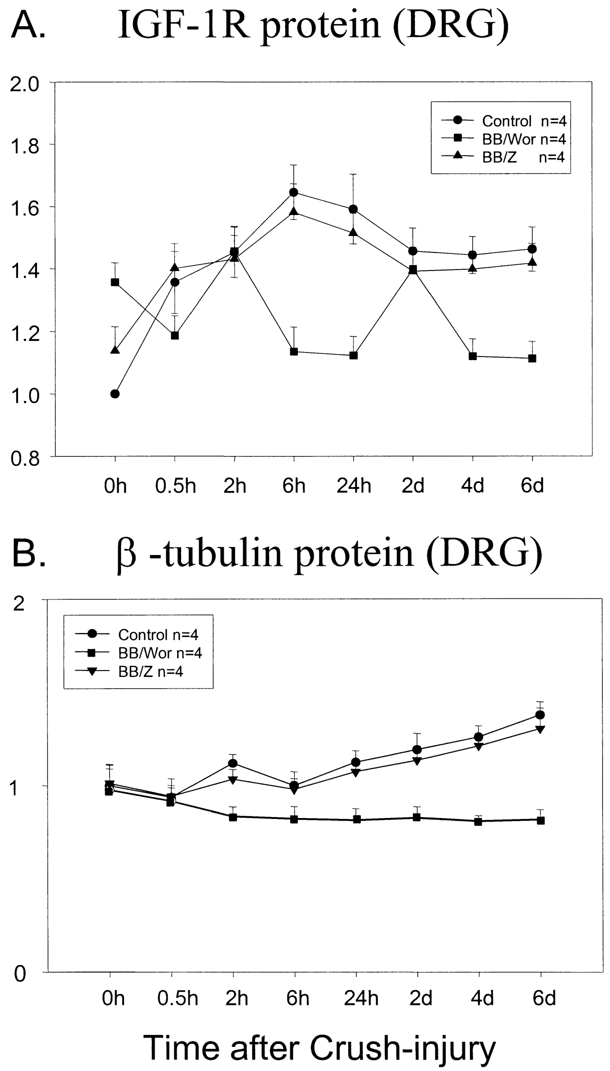

**FIGURE 3**

Protein expression of IGF-IR in dorsal root ganglia from type 1 diabetic BB/Wor and type 2 diabetic BB/Z rats at various time points after sciatic nerve crush injury (*A*). At baseline time point 0 in uninjured 6-week diabetic rats, the IGF-IR expression was significantly ($P < .001$) up-regulated in type 1 rats but not in type 2. Following crush-injury, type 2 diabetic rats showed an immediate upregulation of IGF-IR not significantly different from that of age-matched control animals, whereas the expression in type 1 rats showed an undulating and suppressed profile. Two-way ANOVA showed $P < .001$ ($F = 303$) for time and $P < .001$ ($F = 60$) for Group × Time. This corresponded to a markedly suppressed expression of $\beta$-tubulin in type 1 diabetic rats (*B*), which from 24 hours post crush injury was significantly ($P < .001$) lower than in both control and type 2 BB/Z rats. Instead, isohyperglycemic and normoinsulinemic type 2 rats showed a normal up-regulation of $\beta$-tubulin. Reproduced with permission from *Journal of Neuropathology and Experimental Neurology*.

1993; Kanje et al., 1989). However, other ligands of the IGF family, such as insulin itself, as well as C-peptide, may in part account for these differences in the expression of neuroskeletal proteins. Insulin has neurotrophic effects on peripheral nerve by stabilizing neurofilament and tubulin gene expression in a dose-dependent manner (Fernyhough et al., 1993; Wang et al., 1992), and is required for NGF to exert its protective effects on human neuroblastoma cells (Recio-Pinto and Ishii, 1984). Similar effects have been demonstrated for C-peptide (Li et al., 2001; Pierson et al., 2003; Sima et al., 2002), and are probably mediated via C-peptide's insulinomimetic effects (Grunberger et al., 2001).

A preserved integrity of the IGF system is a requirement for normal nerve regeneration (Chiarelli et al., 2000; Ishii, 1995; Pu et al., 1999; Stoll and Muller, 1999; Xu and Sima, 2001). Nerve fiber regeneration is a spatiotemporally regulated multistep process and a complex interplay between Schwann cells, macrophages, fibroblasts, and neuronal elements (Xu and Sima, 2001). Nerve damage induces early gene responses, which include the first wave of neurotrophic factors by Schwann cells, followed by macrophage recruitment and Wallerian degeneration, interleukin induction, Schwann cell proliferation, and the second wave of neurotrophic factor production (Xu and Sima, 2001). This cascade of interdependent events foster the synthesis of neuronal cytoskeletal elements that initiate and sustain axonal regrowth and regenerations. The early gene responses of trophic factors include the sequential up-regulation of IGF-I, c-fos, and NGF. In type 1 insulinopenic BB/Wor rat, this sequence of events is markedly delayed and suppressed in its magnitude (Pierson et al., 2002b; Xu and Sima, 2001), following sciatic nerve crush injury (Figure 3). These abnormalities are subsequently associated with perturbed expression of neurofilaments and tubulins and impaired fiber regeneration. Interestingly in the normoinsulinemic but isohyperglycemic type 2 BB/Z rat, these abnormalities do not occur or are substantially milder (Pierson et al., 2002b) and are prevented in the type 1 model following C-peptide replacement (Pierson et al., 2003), followed by normalization of neuroskeletal protein expression (Figure 4). These findings suggest that the perturbed regeneration in type 1 diabetes is not likely to be the result of hyperglycemia per se, but rather the consequence of insulin and/or C-peptide deficiency. This construct is also reflected by a close to normal nerve regeneration in the type 2 model and in C-peptide–replaced type 1 rats, the latter most likely mediated by C-peptide's insulin-enhancing effect (Grunberger et al., 2001).

Further effects of C-peptide replacement include normalization of the expression of IGF-I, IGF-IR, and the IR in peripheral nerve of type 1 BB/Wor rat (Sima et al., 2001a), which may be mediated via its effect on NF-$\kappa$B (Zhang et al., 2002).

One of the most profound differences between DPN in both human and experimental type 1 and type 2 DPN is the progressive nodal and paranodal changes occurring in the former (Sima et al., 1988; Sugimoto et al., 2000a). These changes consist of disruption of the paranodal barrier (axo-glial dysjunction) and migration of crucial sodium channels away from the nodal gap (Cherian et al., 1996; Sima et al., 1986, 1988), and account for the more severe nerve conduction defect in type 1 DPN.

Interestingly, the insulinomimetic C-peptide prevents these changes in type 2 BB/Wor rats (Sima et al., 2000, 2001b), which we have suggested may be accounted for by the colocalization of the IR to axo-glial junctions of the paranodal apparatus and the nodal axolemma (Sugimoto et al., 2000b, 2002). Preliminary studies in our laboratory have shown that the expression or post-translational modifications of several key molecules of the paranodal apparatus, such as caspr, contactin, ankyrin$_G$, and sodium channel $\beta$ subunits of the nodal axolemma, are significantly perturbed in type 1 BB/Wor rats but not in type 2 BB/Z rats, and that these abnormalities can be largely prevented by C-peptide (Sima et al., 2001b, unpublished data). These data strongly suggest that deficiencies of insulin itself and its "helper" C-peptide play important roles in the pathogenesis of type 1 DPN and are likely to be involved in the downstream gene regulation of several members of the IGF system, and that C-peptide replacement in this type of diabetes mellitus may provide a significant therapeutic potential in type 1 DPN.

*In Vitro Studies*

The effects of IGF on diabetic neuropathy have been studies in vitro in cultured superior cervical ganglia (SCG) and DRG neurons, Schwann cells, and neuroblastoma SH-SY5Y cells exposed to high concentrations of glucose or mannitol. High glucose inhibits neurite outgrowth and initiates apoptosis via activation of caspase-3 in cultured SCG and DRG neurons. The addition of IGF-I ameliorates these changes and prevents activation of caspase-3 and neuronal apoptosis (Russell et al., 1999). Similarly, Schwann cells cultured in high glucose undergo apoptosis, which is prevented by IGF-I via PI 3-kinase activation (Delaney et al., 2001).

The neuroblastoma SH-SY5Y cell system is a well-characterized in vitro model for studying neuronal development and apoptosis. Numerous studies have demonstrated that high glucose/mannitol–induced apoptosis of SH-SY5Y cells can be rescued by IGF-I (Cheng and Feldman, 1998; Matthews and Feldman, 1996; Singleton et al., 1996; van Golen et al., 2000; van Golen and Feldman, 2000). Recently, we demonstrated that glucose-induced apoptosis in SH-SY5Y cells is dose and time dependent. To separate the effect of glucose toxicity from that of hyperosmolarity, we also examined the effects of the same concentrations of mannitol on SH-SY5Y cell apoptosis.

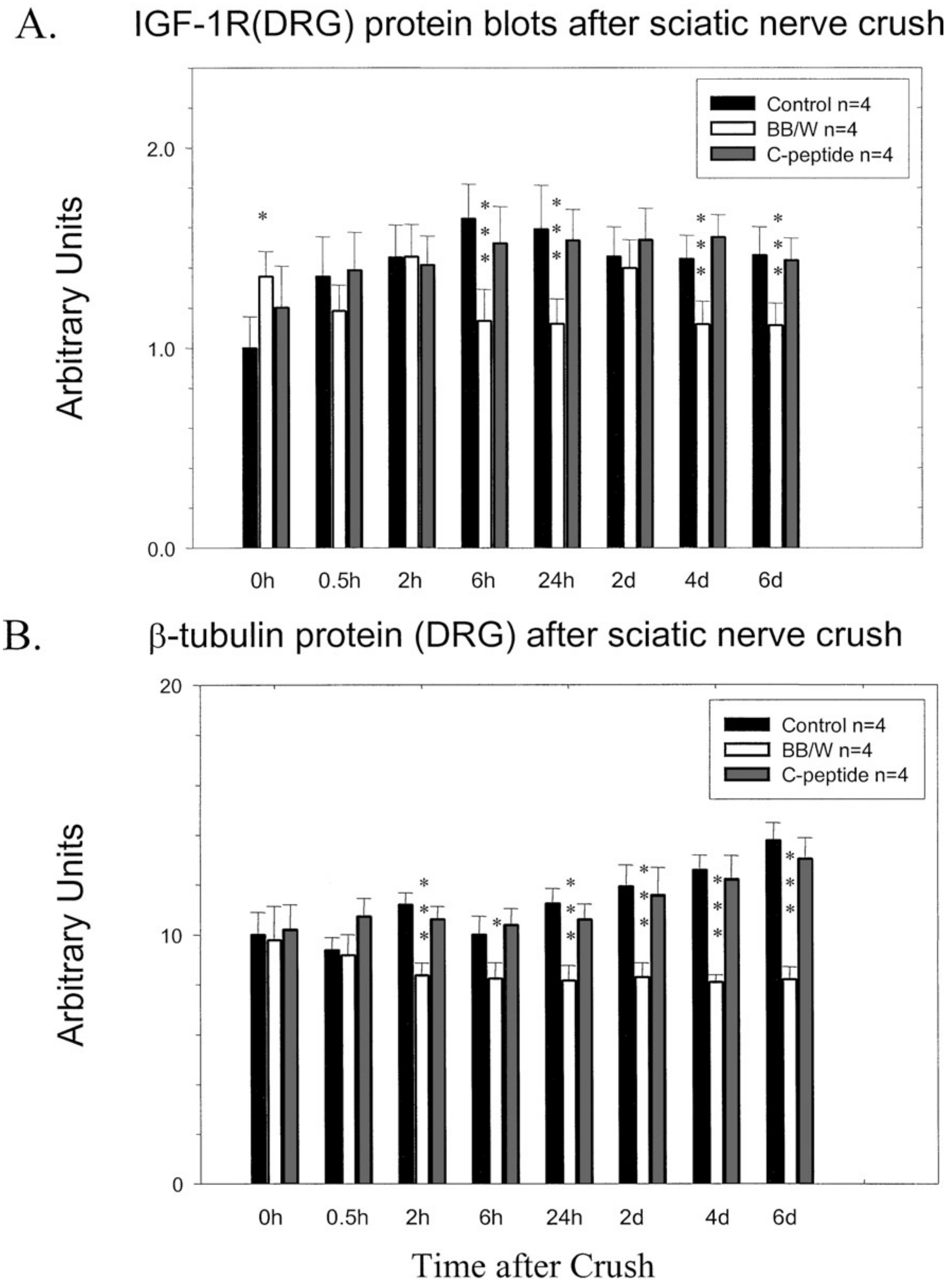

**FIGURE 4**

Protein expression of IGF-IR in dorsal root ganglia in type 1 diabetic BB/Wor rat and those replaced with C-peptide (*A*). C-peptide replacement from onset of diabetes (75 nmol/kg body weight/day) resulted in a normalization of the IGF-IR expression following sciatic nerve crush injury induced at 6 weeks of diabetes. This was accompanied by a normal expression profile of $\beta$-III tubulin (*B*), demonstrating that C-peptide has beneficial and normalizing effects on cytoskeletal protein expression in regenerating sensory nerve fibers in type 1 diabetes.

The extent of apoptosis induced by mannitol was significantly less than that induced by same concentration of glucose (except for extremely high concentration), suggesting that glucose toxicity per se is additive to the hyperosmolar apoptotic effects.

Our data showed that insulin (4 nM) significantly increased neurite outgrowth of SH-SY5Y cells and decreased apoptosis under high glucose concentrations. The addition of C-peptide to insulin showed further significant increases in neurite outgrowth and prevention of apoptosis. The combination of C-peptide and insulin enhanced phosphorylation of IR, but not IGF-IR, suggesting that insulin and C-peptide may signal via a common pathway. On the other hand, IGF-I showed the greatest effect on neurite outgrowth; however, the combination of IGF-I and C-peptide did not result in an additive effect on neurite outgrowth (Zhang et al., 2001, 2002).

In summary, it is well established that abnormalities of IGFs play central roles in the pathogenesis of DPN and that they impair axonal integrity and nerve fiber regeneration in DPN. These effects appear to be most expressed in type 1 diabetes, suggesting that factors other than hyperglycemia are at play. Data from our laboratory, using the proinsulin C-peptide as an insulinomimeticum, strongly suggest that insulin deficiency per se may be the major underlying culprit in the various perturbations of the IGF system. We therefore suggest that C-peptide, like insulin, be incorporated as one of the key ligands in the IGF system.

The clinical usage of IGF-I as a therapeutic agent is probably associated with several problems such as route of administration and its ubiquitous effects with potential adverse effects. Nevertheless, there are some short-term beneficial effects reported in both type 1 and type 2 patients with recombinant human IGF-I (Thrailkill, 2000). However, long-term efficacy data are lacking. Probably, a more applicable and safer approach would be to secondarily modify and correct the IGF system, particularly in type 1 diabetes, via replacement of C-peptide for instance (Sima et al., 2001b).

### CNS COMPLICATION

Recent studies have provided substantial evidence that diabetes has unique impacts on the CNS. Diabetes increases stroke risk, affects glucose transport, and alters the blood-brain barrier (McCall, 1992; Mooradian, 1997). Studies in clinical and experimental diabetes suggest a variety of abnormalities in brain structure, neurotransmitters, and electrophysiology (Dejgaard et al., 1991; Jakobsen et al., 1987; Kamijo et al., 1993; Lackovic et al., 1990; Luse, 1970; Salkovic et al., 1995; Sima et al., 1992; Welsh and Wecker, 1991). The incidence of psychiatric disorders are more common in diabetic patients compared with age-matched nondiabetic subjects (Popkin, 1997), such as major depression and phobias (Pozzessere et al., 1988). Cognitive deficits have been documented in diabetic patients, including memory retention/retrieval and complex reasoning skills (Gispen and Biessels, 2000; Helkala et al., 1995; Mooradian et al., 1988; Popkin et al., 1988; Pozzessere et al., 1988, 1991). The reasons for this may be multifold, such as a higher incidence of stroke, which also behaves differently in diabetic patients (“stroke in evolution”) (Asplund et al., 1980; Fisher and Garcia, 1996; Raev, 1993; Weinberger et al., 1983), arteriolosclerosis with cortical microinfarctions (Dolman, 1963; Reske-Nielsen et al., 1965), and/or diffuse white matter lesions (“leucoariosis”) (Pantoni and Garcia, 1997). These changes are more common in elderly type 2 patients and are accentuated by hypertension. In contrast, repeated episodes of hypoglycemia in type 1 patients are known to cause neuronal loss in the hippocampus, cortical layers 3 and 5, and cerebellar Purkinje cells (Agardh et al., 1981; Auer et al., 1984a, 1984b), with ensuing cognitive and neurological deficits. However, Kramer et al. (1998) and Schoenle et al. (2002) reported that type 1 diabetic patients show a duration-dependent decline in cognitive function, which is unrelated to hypoglycemic episodes. Therefore, cognitive deficiency may be caused by diabetes per se. Taken together, mounting evidence support the notion that the brain is not spared from the complications of diabetes. In diabetic rats, reduction of neocortical thickness associated with neuronal loss (Jakobsen et al., 1987; Luse, 1970), decreased acetylcholine synthesis and release (Welsh and Wecker, 1991), impaired spatial memory and learning (Biessels et al., 1996; Popovic et al., 2001), and altered synaptic integrity of the hippocampus as reflected by long-term potentiation deficits (Biessels et al., 1998; Kamal et al., 2000) have been demonstrated. These animal studies also suggest that a primary encephalopathy occurs in diabetes.

The mechanisms by which diabetes causes CNS complications are not clear. Hippocampus and frontal cortex are essential parts of the limbic system and are intimately correlated with higher cognitive functions. They are vulnerable parts of the brain, with high susceptibility to ischemia and hypoglycemia. In CNS, apoptotic neuronal cell death has been described in ischemic brain injury (Kihara et al., 1994; Li et al., 1995; Linnik et al., 1993; Nitatori et al., 1995) and in neurodegenerative diseases, such as Alzheimer disease, Parkinson’s disease, Huntington’s disease, and amyotrophic lateral sclerosis (Hartley et al., 1994; Loo et al., 1993; Offen et al., 1999; Stefanis et al., 1997; Tatton et al., 1997, 1998; Ziv and Melamed, 1998). We recently reported on a duration-dependent neuronal apoptosis in hippocampus of type 1 diabetic BB/Wor rats, which is accompanied with neuronal loss and cognitive impairments. As shown in Figure 5, perturbations of the IGF system was

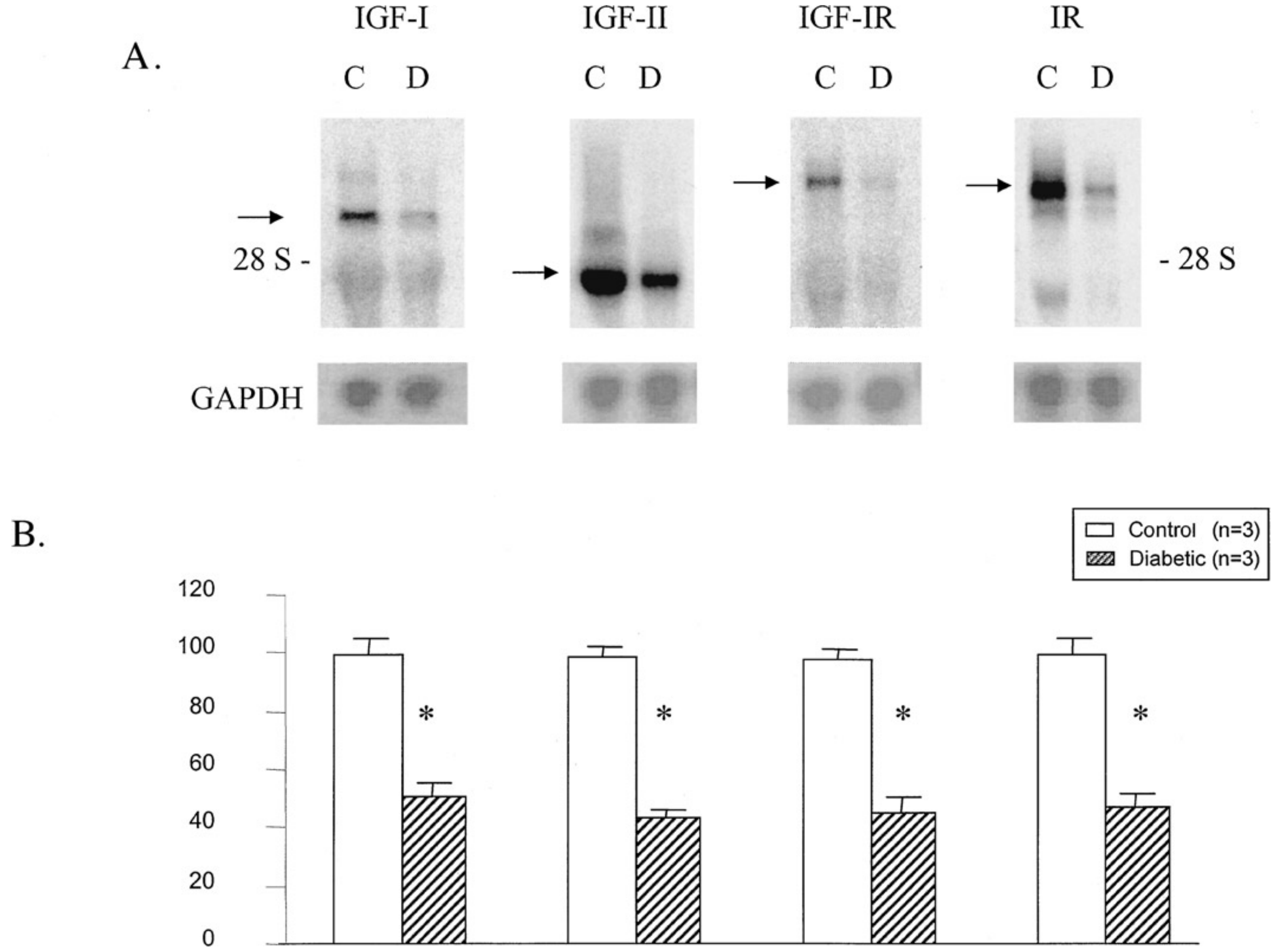

**FIGURE 5**
Perturbations of the IGF system in CNS in type 1 diabetic BB/Wor-rat. (*A*) Northern blot hybridization (representative of three blots). The mRNA levels of and IGF-I, IGF-II, IGF-IR, and IR were significantly reduced in hippocampi of 2-month diabetic BB/Wor rats compared to age-matched control rats (*B*). Control values are arbitrarily set to 100. Position of 28s ribosome is indicated. Arrows indicate the mRNA bands of IGF-I (7.5 kb), IGF-II (3.8 kb), IGF-IR (11 kb), and IR (11 kb) in (*A*). The GAPDH bands of the corresponding gel show that equal amounts of RNA was loaded onto each lane. C, control; D, diabetic. $^{*}P < .001$ versus control. Reproduced with permission from *Brain Research*.

demonstrated prior to the development of hippocampal apoptosis in the BB/Wor rat (Li et al., 2002a). These data suggest that neuronal apoptosis in the diabetic animals is likely to contribute to the cognitive deficiencies observed in type 1 diabetic patients. It is of interest to note that in Alzheimer disease, both IR and IGF-IR are significantly down-regulated in hippocampus (Terry et al., 2001).

### IGF and the CNS Complications of Diabetes in Experimental Animals

Ishii (1995) initially proposed that decreased IGF activities may contribute to CNS alterations in diabetes. This hypothesis has subsequently been supported by a series of clinical and experimental studies. Many studies show that IGF activity is impaired in the CNS of experimental diabetic animals. The expression of IGF-I and IGF-II are reduced in the spinal cord and brain of type 1 diabetic STZ and BB/Wor rats (Busiguina et al., 1996; Wuarin et al., 1994; Zhuang et al., 1997; Li et al., 2002a, 2002b) and in the spontaneously diabetic ZDF(fa/fa) (Wuarin et al., 1996). Diminished IGF-II gene expression in the brain precedes abnormalities in brain structure, neurotransmitter levels, $Na^{+}$,$K^{+}$-ATPase activity and learning behaviors in STZ-induced diabetes (Wuarin et al., 1996), suggesting that alterations of the IGF system exerts an important role in the pathogenesis of CNS complications. We have used a model of type 1 diabetes, the spontaneous type 1 diabetic BB/Wor rat, to examined CNS alterations of the IGF system. In this model, IGF-I, IGF-II, IGF-IR, and IR expression was examined in 2- and 8-month diabetic animals, representing an early and a chronic state of diabetes. In both 2-month and 8-month diabetic animals, the expression of IGF-I and IGF-II, IGF-IR, and IR were significantly reduced compared to age-matched control rats, suggesting that the IGF system is impaired at the early stage and continue to decline with the disease progression (Li et al., 2002a). The early impairments of the IGF system preceded significant apoptosis and neuronal cell loss in the $CA_1$ region of the hippocampus, which became evident only at 8-month duration of diabetes. Apoptotic activity correlated with deoxylnucleotidyl transferase-mediated dUTP nick-end labeling (TUNEL) staining, DNA laddering, caspase-3 activity, and $Bax/Bcl_x$ expression. Spatial learning and memory were significantly impaired in diabetic animals (Li et al., 2002a). These results indicate a duration-dependent IGF-associated neuronal apoptosis leading to cognitive dysfunction. Interestingly, C-peptide replacement corrected partially but significantly the abnormalities in IGF-I, IGF-II, IGF-IR, and IR expression in the hippocampus and partially prevented hippocampal apoptosis, neuronal loss, and the functional cognitive impairment (Li et al., 2002b).

In STZ-diabetic rats, insulin treatment prevents water maze learning and hippocampal impairments of long-term potentiation (Biessels et al., 1998), and reverses the prolonged latencies of auditory and visual evoked potentials (Biessels, 1999). These results suggest that insulin deficiency may play a crucial role in impaired CNS function in type 1 diabetes. Because insulin replacement therapy partially restores IGF-II mRNA levels in the cerebral cortex and spinal cord in the same animal model (Wuarin et al., 1996), insulin may exert its role via activation of the IGF system, which would be consistent with the same effects of the insulinomimetic C-peptide.

### In Vitro Studies

In cerebrocortical and cerebellar granule cell cultures, IGF-I significantly reduces cell death induced by glucose deprivation (Harper et al., 1996). Two IGF analogues: QAYL and B-chain mutants, which show reduced affinity for IGFBPs, are as effective as IGF-I in promoting cell survival in conditions of glucose deprivation, indicating that IGF-I promotes neuronal survival. Apoptosis of cerebellar granule cells is blocked by IGF-I (Galli et al., 1995). Preliminary data from our laboratory suggest that primary hippocampal neurons grown in high glucose are protected from apoptosis by either IGF-I, insulin, or C-peptide (Li et al., unpublished data).

### DIFFERENCE IN IGF ACTIVITIES IN THE CNS AND PERIPHERAL NERVE SYSTEM (PNS)

The alterations in IGF activities differ in peripheral nerve from those in the central nervous system in both STZ-diabetic rats and BB/Wor rats. The neurophysiological abnormalities of peripheral nerve usually occur more rapidly than those of the central nervous system (Biessels, 1999; Kamijo et al., 1993; Li et al., 2002b; Sima et al., 1992).

The patterns of decline in the IGF system is different in PNS and CNS in type 1 diabetic BB/Wor rats. IGF perturbations are established in peripheral nerves at 6-week duration of diabetes, whereas this is only evident at 2-month duration of diabetes in the hippocampus. Furthermore, in CNS of diabetic BB/Wor rats, IGF-I, IGF-II, IGF-IR, and IR are decreased at both the mRNA and protein levels (Li et al., 2002a), whereas in the peripheral nerve of the same model, IGF-I and IGF-II expression is reduced, but IR and IGF-IR expression are increased (Sugimoto et al., 2000b; Xu and Sima, 2001). These data suggest that insulin deficiency may involve different mechanisms by which the IGF system is regulated in the PNS versus the CNS.

### CONCLUSIONS AND FUTURE DEVELOPMENTS

More than 15 years of fairly extensive experimental research and limited clinical research have established the IGF system and its perturbations as important pathogenetic factors in the development of the neurological complications, particularly those associated with type 1 diabetes. There is strong evidence to suggest that abnormalities in IGF metabolism occur independently of hyperglycemia and that they are linked to insulinopenia. Limited clinical trials have indicated that restoration of IGF activities may prevent or even to some extent reverse neurological deficits associated with diabetes. However, the ubiquity of IGF activities poses a challenge to target specific tissues, even within the nervous system, because the perturbations of various gene responses of IGFs in the CNS and the PNS divert in opposite directions.

There is evidence to suggest that insulin and the proinsulin C-peptide play important roles in regulating and correcting the diverse aberrations of IGFs. Hence, a logical conjecture of these relationships would be to replenish the insulinomimetic C-peptide in parallel to optimal insulin treatment. In fact several short-term clinical trials have underlined the beneficial effects of C-peptide on several chronic complications, which are in keeping with the experimental data. However, a major obstacle and challenge is to find support for large scale Food and Drug Administration (FDA)-approved clinical trials testing the efficacy of C-peptide in diabetic complications. Because patents on C-peptide are long expired, the incentives on part of the industry to provide the peptide and funds for necessary trials are nonexisting. This stumbling block needs to be overcome by National Institutes of Health (NIH) mechanisms or by support from nonprofit organizations to initiate appropriately designed clinical trials. A potential but distant possibility is the developments within the human genome research projects targeting not only IGF-I itself, but also related peptides such as C-peptide or others. However, these possibilities are to the best of the knowledge not yet in the plans.

### REFERENCES

Agardh, C. D., Kalimo, H., Olsson, Y., and Siesjö, B. K. (1981) Hypoglycemic brain injury: Metabolic and structural findings in rat cerebellar cortex during profound insulin-induced hypoglycemia and in the recovery period following glucose administration. *J. Cereb. Blood Flow Metab.,* **1,** 71–84.

Aikawa, R., Nawano, M., Gu, Y., Katagiri, H., Asano, T., Zhu, W., Nagai, R., and Komuro, I. (2000) Insulin prevents cardiomyocytes from oxidative stress-induced apoptosis through activation of PI3 kinase/Akt. *Circulation,* **102,** 2873–2879.

Alexi, T., Hughes, P. E., van Roon-Mom, W. M., Faull, R. L., Williams, C. E., Clark, R. G., and Gluckman, P. D. (1999) The IGF-I amino-terminal tripeptide glycine-proline-glutamate (GPE) is neuroprotective to striatum in the quinolinic acid lesion animal model of Huntington's disease. *Exp. Neurol.,* **159,** 84–97.

Ang, L. C., Bhaumick, B., Munoz, D. G., Sass, J., and Juurlink, B. H. (1992) Effects of astrocytes, insulin and insulin-like growth factor I on the survival of motoneurons in vitro. *J. Neurol. Sci.,* **109,** 168–172.

Arner, P., Sjöberg, S., Gjotterberg, M., and Skottner, A. (1989) Circulating insulin-like growth factor I in type 1 (insulin-dependent) diabetic patients with retinopathy. *Diabetologia,* **32,** 753–758.

Arsenijevic, Y., and Weiss, S. (1998) Insulin-like growth factor-I is a differentiation factor for postmitotic CNS stem cell-derived neuronal precursors: Distinct actions from those of brain-derived neurotrophic factor. *J. Neurosci.,* **18,** 2118–2128.

Asplund, K., Hägg, E., Helmers, C., Lithner, F., Strand, T., and Wester, P. O. (1980) The natural history of stroke in diabetic patients. *Acta Med. Scand.,* **207,** 417–424.

Auer, R. N., Olsson, Y., and Siesjö, B. K. (1984a) Hypoglycemic brain injury in the rat. Correlation of density of brain damage with the EEG isoelectric time: A quantitative study. *Diabetes,* **33,** 1090–1098.

Auer, R. N., Wieloch, T., Olsson, Y., and Siesjo, B. K. (1984b) The distribution of hypoglycemic brain damage. *Acta Neuropathol. (Berl.),* **64,** 177–191.

Avruch, J., Törnqvist, H. E., Gunsalus, J. R., Price, D. J., Kyriakis, J. M., Yurkow, E. J., Ahmad, M. F., and Banerjee, P. (1990) The role of tyrosine- and serine-threonine-protein phosphorylation in insulin action. *Adv. Second Messenger Phosphoprotein Res.,* **24,** 295–300.

Bai, H., Pollman, M. J., Inishi, Y., and Gibbons, G. H. (1999) Regulation of vascular smooth muscle cell apoptosis. Modulation of bad by a phosphatidylinositol 3-kinase-dependent pathway. *Circ. Res.,* **85,** 229–237.

Baxter, R. C. (1994) Insulin-like growth factor binding proteins in the human circulation: A review. *Horm. Res.,* **42,** 140–144.

Baxter, R. C. (2000) Insulin-like growth factor (IGF)-binding proteins: Interactions with IGFs and intrinsic bioactivities. *Am. J. Physiol. Endocrinol. Metab.,* **278,** E967–E976.

Baxter, R. C. (2001) Changes in the IGF-IGFBP axis in critical illness. *Best Pract. Res. Clin. Endocrinol. Metab.,* **15,** 421–434.

Bence-Hanulec, K. K., Marshall, J., and Blair, L. A. (2000) Potentiation of neuronal L calcium channels by IGF-1 requires phosphorylation of the alpha1 subunit on a specific tyrosine residue. *Neuron,* **27,** 121–131.

Bereket, A., Lang, C. H., Blethen, S. L., Ng, L. C., and Wilson, T. A. (1996) Insulin treatment normalizes reduced free insulin-like growth factor-I concentrations in diabetic children. *Clin. Endocrinol. (Oxf.),* **45,** 321–326.

Bertrand, F., Atfi, A., Cadoret, A., L'Allemain, G., Robin, H., Lascols, O., Capeau, J., and Cherqui, G. (1998) A role for nuclear factor kappaB in the antiapoptotic function of insulin. *J. Biol. Chem.,* **273,** 2931–2938.

Bertrand, F., Desbois-Mouthon, C., Cadoret, A., Prunier, C., Robin, H., Capeau, J., Atfi, A., and Cherqui, G. (1999) Insulin antiapoptotic signaling involves insulin activation of the nuclear factor kappaB-dependent survival genes encoding tumor necrosis factor receptor-associated factor 2 and manganese-superoxide dismutase. *J. Biol. Chem.,* **274,** 30596–30602.

Biessels, G. J. (1999) Cerebral complications of diabetes: Clinical findings and pathogenetic mechanisms. *Neth. J. Med.,* **54,** 35–45.

Biessels, G. J., Kamal, A., Ramakers, G. M., Urban, I. J., Spruijt, B. M., Erkelens, D. W., and Gispen, W. H. (1996) Place learning and hippocampal synaptic plasticity in streptozotocin-induced diabetic rats. *Diabetes,* **45,** 1259–1266.

Biessels, G. J., Kamal, A., Urban, I. J., Spruijt, B. M., Erkelens, D. W., and Gispen, W. H. (1998) Water maze learning and hippocampal synaptic plasticity in streptozotocin-diabetic rats: Effects of insulin treatment. *Brain Res.,* **800,** 125–135.

Bitar, M. S., Pilcher, C. W., Khan, I., and Waldbillig, R. J. (1997) Diabetes-induced suppression of IGF-1 and its receptor mRNA levels in rat superior cervical ganglia. *Diabetes Res. Clin. Pract.,* **38,** 73–80.

Blair, L. A., Bence-Hanulec, K. K., Mehta, S., Franke, T., Kaplan, D., and Marshall, J. (1999) Akt-dependent potentiation of L channels by insulin-like growth factor-1 is required for neuronal survival. *J. Neurosci.,* **19,** 1940–1951.

Blair, L. A., and Marshall, J. (1997) IGF-1 modulates N and L calcium channels in a PI 3-kinase-dependent manner. *Neuron,* **19,** 421–429.

Blakesley, V. A., Scrimgeour, A., Esposito, D., and Le Roith, D. (1996) Signaling via the insulin-like growth factor-I receptor: Does it differ from insulin receptor signaling? *Cytokine Growth Factor Rev.,* **7,** 153–159.

Blundell, T. L., Bedarkar, S., and Humbel, R. E. (1983) Tertiary structures, receptor binding, and antigenicity of insulinlike growth factors. *Fed. Proc.,* **42,** 2592–2597.

Bonni, A., Brunet, A., West, A. E., Datta, S. R., Takasu, M. A., and Greenberg, M. E. (1999) Cell survival promoted by the Ras-MAPK signaling pathway by transcription-dependent and -independent mechanisms. *Science,* **286,** 1358–1362.

Bornfeldt, K. E., Arnqvist, H. J., Enberg, B., Mathews, L. S., and Norstedt, G. (1989) Regulation of insulin-like growth factor-I and growth hormone receptor gene expression by diabetes and nutritional state in rat tissues. *J. Endocrinol.,* **122,** 651–656.

Brooker, G. J., Kalloniatis, M., Russo, V. C., Murphy, M., Werther, G. A., and Bartlett, P. F. (2000) Endogenous IGF-1 regulates the neuronal differentiation of adult stem cells. *J. Neurosci. Res.,* **59,** 332–341.

Brownawell, A. M., Kops, G. J., Macara, I. G., and Burgering, B. M. (2001) Inhibition of nuclear import by protein kinase B (Akt) regulates the subcellular distribution and activity of the forkhead transcription factor AFX. *Mol. Cell. Biol.,* **21,** 3534–3546.

Brunet, A., Bonni, A., Zigmond, M. J., Lin, M. Z., Juo, P., Hu, L. S., Anderson, M. J., Arden, K. C., Blenis, J., and Greenberg, M. E. (1999) Akt promotes cell survival by phosphorylating and inhibiting a Forkhead transcription factor. *Cell,* **96,** 857–868.

Buckley, D. A., Cheng, A., Kiely, P. A., Tremblay, M. L., and O'Connor, R. (2002) Regulation of insulin-like growth factor type I (IGF-I) receptor kinase activity by protein tyrosine phosphatase 1B (PTP-1B) and enhanced IGF-I-mediated suppression of apoptosis and motility in PTP-1B-deficient fibroblasts. *Mol. Cell. Biol.,* **22,** 1998–2010.

Busiguina, S., Chowen, J. A., Argente, J., and Torres-Aleman, I. (1996) Specific alterations of the insulin-like growth factor I system in the cerebellum of diabetic rats. *Endocrinology,* **137,** 4980–4987.

Busiguina, S., Fernandez, A. M., Barrios, V., Clark, R., Tolbert, D. L., Berciano, J., and Torres-Aleman, I. (2000) Neurodegeneration is associated to changes in serum insulin-like growth factors. *Neurobiol. Dis.,* **7,** 657–665.

Cano, E., Doza, Y. N., Ben-Levy, R., Cohen, P., and Mahadevan, L. C. (1996) Identification of anisomycin-activated kinases p45 and p55 in murine cells as MAPKAP kinase-2. *Oncogene,* **12,** 805–812.

Cheng, B., and Mattson, M. P. (1992) IGF-I and IGF-II protect cultured hippocampal and septal neurons against calcium-mediated hypoglycemic damage. *J. Neurosci.,* **12,** 1558–1566.

Cheng, H. L., and Feldman, E. L. (1997) Insulin-like growth factor-I (IGF-I) and IGF binding protein-5 in Schwann cell differentiation. *J. Cell. Physiol.,* **171,** 161–167.

Cheng, H. L., and Feldman, E. L. (1998) Bidirectional regulation of p38 kinase and c-Jun N-terminal protein kinase by insulin-like growth factor-I. *J. Biol. Chem.,* **273,** 14560–14565.

Cheng, H. L., Russell, J. W., and Feldman, E. L. (1999) IGF-I promotes peripheral nervous system myelination. *Ann. N.Y. Acad. Sci.,* **883,** 124–130.

Cherian, P. V., Kamijo, M., Angelides, K. J., and Sima, A. A. F. (1996) Nodal Na(+)-channel displacement is associated with nerve-conduction slowing in the chronically diabetic BB/W rat: Prevention by aldose reductase inhibition. *J. Diabetes Complications,* **10,** 192–200.

Chiarelli, F., Santilli, F., and Mohn, A. (2000) Role of growth factors in the development of diabetic complications. *Horm. Res.,* **53,** 53–67.

Chou, C. K., Dull, T. J., Russell, D. S., Gherzi, R., Lebwohl, D., Ullrich, A., and Rosen, O. M. (1987) Human insulin receptors mutated at the ATP-binding site lack protein tyrosine kinase activity and fail to mediate postreceptor effects of insulin. *J. Biol. Chem.,* **262,** 1842–1847.

Cinaz, P., Kendirci, M., Kurtoglu, S., Gokcora, N., Buyan, N., Yavuz, I., and Demir, A. (1996) Serum levels of insulin-like growth factor-I and insulin-like growth factor binding protein-3 in children with insulin-dependent diabetes mellitus. *J. Pediatr. Endocrinol. Metab.,* **9,** 475–482.

Clauson, P. G., Brismar, K., Hall, K., Linnarsson, R., and Grill, V. (1998) Insulin-like growth factor-I and insulin-like growth factor binding protein-1 in a representative population of type 2 diabetic patients in Sweden. *Scand. J. Clin. Lab. Invest.,* **58,** 353–360.

Clemmons, D. R., Busby, W., Clarke, J. B., Parker, A., Duan, C., and Nam, T. J. (1998) Modifications of insulin-like growth factor binding proteins and their role in controlling IGF actions. *Endocr. J.,* **45(Suppl),** S1–S8.

Copelman, C. A., Cuzner, M. L., Groome, N., and Diemel, L. T. (2000) Temporal analysis of growth factor mRNA expression in myelinating rat brain aggregate cultures: Increments in CNTF, FGF-2, IGF-I, and PDGF-AA mRNA are induced by antibody-mediated demyelination. *Glia,* **30,** 342–351.

Craner, M. J., Klein, J. P., Black, J. A., and Waxman, S. G. (2002) Preferential expression of IGF-I in small DRG neurons and down-regulation following injury. *Neuroreport,* **13,** 1649–1652.

Crosby, S. R., Tsigos, C., Anderton, C. D., Gordon, C., Young, R. J., and White, A. (1992) Elevated plasma insulin-like growth factor binding protein-1 levels in type 1 (insulin-dependent) diabetic patients with peripheral neuropathy. *Diabetologia,* **35,** 868–872.

The DCCT Research Group. (1993) The effect of intensive treatment of diabetes on the development and progression of long-term complications in insulin-dependent diabetes mellitus. The Diabetes Control and Complications Trial Research Group. *N. Engl. J. Med.,* **329,** 977–986.

De La Puente, A., Goya, L., Ramos, S., Martin, M. A., Alvarez, C., Escriva, F., and Pascual-Leone, A. M. (2000) Effects of experimental diabetes on renal IGF/IGFBP system during neonatal period in the rat. *Am. J. Physiol. Renal. Physiol.,* **279,** F1067–F1076.

Dejgaard, A., Gade, A., Larsson, H., Balle, V., Parving, A., and Parving, H. H. (1991) Evidence for diabetic encephalopathy. *Diabet. Med.,* **8,** 162–167.

Delaney, C. L., Russell, J. W., Cheng, H. L., and Feldman, E. L. (2001) Insulin-like growth factor-I and over-expression of Bcl-xL prevent glucose-mediated apoptosis in Schwann cells. *J. Neuropathol. Exp. Neurol.,* **60,** 147–160.

D'Ercole, A. J., Ye, P., Calikoglu, A. S., and Gutierrez-Ospina, G. (1996) The role of the insulin-like growth factors in the central nervous system. *Mol. Neurobiol.,* **13,** 227–255.

D'Mello, S. R., Borodezt, K., and Soltoff, S. P. (1997) Insulin-like growth factor and potassium depolarization maintain neuronal survival by distinct pathways: Possible involvement of PI 3-kinase in IGF-1 signaling. *J. Neurosci.,* **17,** 1548–1560.

Dolman, C. L. (1963) The morbid anatomy of diabetic neuropathy. *Neurology,* **13,** 135–142.

Dore, S., Kar, S., and Quirion, R. (1997) Rediscovering an old friend, IGF-I: Potential use in the treatment of neurodegenerative diseases. *Trends Neurosci.,* **20,** 326–331.

Dubois-Dalcq, M., and Murray, K. (2000) Why are growth factors important in oligodendrocyte physiology? *Pathol. Biol. (Paris),* **48,** 80–86.

Dudek, H., Datta, S. R., Franke, T. F., Birnbaum, M. J., Yao, R., Cooper, G. M., Segal, R. A., Kaplan, D. R., and Greenberg, M. E. (1997) Regulation of neuronal survival by the serine-threonine protein kinase Akt. *Science,* **275,** 661–665.

Dyck, P. J., Davies, J. L., Wilson, D. M., Service, F. J., Melton, L. J., 3rd, and O'Brien, P. C. (1999) Risk factors for severity of diabetic polyneuropathy: Intensive longitudinal assessment of the Rochester Diabetic Neuropathy Study cohort. *Diabetes Care,* **22,** 1479–1486.

Dyck, P. J., Kratz, K. M., Karnes, J. L., Litchy, W. J., Klein, R., Pach, J. M., Wilson, D. M., O'Brien, P. C., Melton, L. J., 3rd, and Service,

F. J. (1993) The prevalence by staged severity of various types of diabetic neuropathy, retinopathy, and nephropathy in a population-based cohort: The Rochester Diabetic Neuropathy Study. *Neurology,* **43,** 817–824.

Ebina, Y., Araki, E., Taira, M., Shimada, F., Mori, M., Craik, C. S., Siddle, K., Pierce, S. B., Roth, R. A., and Rutter, W. J. (1987) Replacement of lysine residue 1030 in the putative ATP-binding region of the insulin receptor abolishes insulin- and antibody-stimulated glucose uptake and receptor kinase activity. *Proc. Natl. Acad. Sci. U. S. A.,* **84,** 704–708.

Ekman, B., Nystrom, F., and Arnqvist, H. J. (2000) Circulating IGF-I concentrations are low and not correlated to glycaemic control in adults with type 1 diabetes. *Eur. J. Endocrinol.,* **143,** 505–510.

Feldman, E. L., Sullivan, K. A., Kim, B., and Russell, J. W. (1997) Insulin-like growth factors regulate neuronal differentiation and survival. *Neurobiol. Dis.,* **4,** 201–214.

Fernyhough, P., Diemel, L. T., Brewster, W. J., and Tomlinson, D. R. (1994) Deficits in sciatic nerve neuropeptide content coincide with a reduction in target tissue nerve growth factor messenger RNA in streptozotocin-diabetic rats: Effects of insulin treatment. *Neuroscience,* **62,** 337–344.

Fernyhough, P., Diemel, L. T., and Tomlinson, D. R. (1998) Target tissue production and axonal transport of neurotrophin-3 are reduced in streptozotocin-diabetic rats. *Diabetologia,* **41,** 300–306.

Fernyhough, P., Willars, G. B., Lindsay, R. M., and Tomlinson, D. R. (1993) Insulin and insulin-like growth factor I enhance regeneration in cultured adult rat sensory neurones. *Brain Res.,* **607,** 117–124.

Fisher, M., and Garcia, J. H. (1996) Evolving stroke and the ischemic penumbra. *Neurology,* **47,** 884–888.

Flores-Riveros, J. R., Sibley, E., Kastelic, T., and Lane, M. D. (1989) Substrate phosphorylation catalyzed by the insulin receptor tyrosine kinase. Kinetic correlation to autophosphorylation of specific sites in the beta subunit. *J. Biol. Chem.,* **264,** 21557–21572.

Foulstone, E. J., Meadows, K. A., Holly, J. M., and Stewart, C. E. (2001) Insulin-like growth factors (IGF-I and IGF-II) inhibit C2 skeletal myoblast differentiation and enhance TNF alpha-induced apoptosis. *J. Cell. Physiol.,* **189,** 207–215.

Fujihara, M., Uemasu, J., and Kawasaki, H. (1996) Serum and urinary levels of insulin-like growth factor I in patients with chronic renal disease and diabetes mellitus: Its clinical implication. *Clin. Nephrol.,* **45,** 372–378.

Galli, C., Meucci, O., Scorziello, A., Werge, T. M., Calissano, P., and Schettini, G. (1995) Apoptosis in cerebellar granule cells is blocked by high KCl, forskolin, and IGF-1 through distinct mechanisms of action: The involvement of intracellular calcium and RNA synthesis. *J. Neurosci.,* **15,** 1172–1179.

Gamper, N., Fillon, S., Huber, S. M., Feng, Y., Kobayashi, T., Cohen, P., and Lang, F. (2002) IGF-1 up-regulates $K^+$ channels via PI3-kinase, PDK1 and SGK1. *Pflugers Arch.,* **443,** 625–634.

Gispen, W. H., and Biessels, G. J. (2000) Cognition and synaptic plasticity in diabetes mellitus. *Trends Neurosci.,* **23,** 542–549.

Glazner, G. W., Lupieu, S., Miller, J. A., and Ishii, D. N. (1993) Insulin-like growth factor-II increases the rate of sciatic nerve regeneration in rats. *Neuroscience,* **54,** 791–797.

Gluckman, P., Klempt, N., Guan, J., Mallard, C., Sirimanne, E., Dragunow, M., Klempt, M., Singh, K., Williams, C., and Nikolics, K. (1992) A role for IGF-1 in the rescue of CNS neurons following hypoxic-ischemic injury. *Biochem. Biophys. Res. Commun.,* **182,** 593–599.

Grandis, M., Nobbio, L., Abbruzzese, M., Banchi, L., Minuto, F., Barreca, A., Garrone, S., Mancardi, G. L., and Schenone, A. (2001) Insulin treatment enhances expression of IGF-I in sural nerves of diabetic patients. *Muscle Nerve,* **24,** 622–629.

Graubert, M. D., Goldstein, S., and Phillips, L. S. (1991) Nutrition and somatomedin. XXVII. Total and free IGF-I and IGF binding proteins in rats with streptozocin-induced diabetes. *Diabetes,* **40,** 959–965.

Greene, D. A., Sima, A. A. F., Feldman, E. L., and Stevens, M. J. (1997) In: *Diabetes Mellitus,* Edited by Rifkin, H., Porte, D., and Sherwin, R., pp. 1009–1076. Stanford, CT, Appleton and Lange.

Greene, D. A., Sima, A. A. F., Stevens, M. J., Feldman, E. L., and Lattimer, S. A. (1992) Complications: Neuropathy, pathogenetic considerations. *Diabetes Care,* **15,** 1902–1925.

Gronborg, M., Wulff, B. S., Rasmussen, J. S., Kjeldsen, T., and Gammeltoft, S. (1993) Structure-function relationship of the insulin-like growth factor-I receptor tyrosine kinase. *J. Biol. Chem.,* **268,** 23435–23440.

Grunberger, G., Xiaoling, Q., Li, Z. G., Mathews, S. T., Sbriessa, D., Shisheva, A., and Sima, A. A. F. (2001) Molecular basis for the insulinomimetic effects of C-peptide. *Diabetologia,* **44,** 1247–1257.

Guan, J., Krishnamurthi, R., Waldvogel, H. J., Faull, R. L., Clark, R., and Gluckman, P. (2000) N-terminal tripeptide of IGF-1 (GPE) prevents the loss of TH positive neurons after 6-OHDA induced nigral lesion in rats. *Brain Res.,* **859,** 286–292.

Guan, J., Waldvogel, H. J., Faull, R. L., Gluckman, P. D., and Williams, C. E. (1999) The effects of the N-terminal tripeptide of insulin-like growth factor-1, glycine-proline-glutamate in different regions following hypoxic-ischemic brain injury in adult rats. *Neuroscience,* **89,** 649–659.

Guan, J., Williams, C., Gunning, M., Mallard, C., and Gluckman, P. (1993) The effects of IGF-1 treatment after hypoxic-ischemic brain injury in adult rats. *J. Cereb. Blood Flow Metab.,* **13,** 609–616.

Guo, H., Yang, Y., Geng, Z., Zhu, L., Yuan, S., Zhao, Y., Gao, Y., and Fu, H. (1999) The change of insulin-like growth factor-1 in diabetic patients with neuropathy. *Chin. Med. J. (Engl.),* **112,** 76–79.

Hall, K. E., Sima, A. A. F., and Wiley, J. W. (1995) Voltage-dependent calcium currents are enhanced in dorsal root ganglion neurones from the Bio Breeding/Worchester diabetic rat. *J. Physiol.,* **486 (Pt 2),** 313–322.

Harper, S. J., Macaulay, A. J., Hill, R. G., and Priestley, T. (1996) The effects of insulin-like growth factor analogues on survival of cultured cerebral cortex and cerebellar granule neurones. *Brain Res.,* **709,** 303–310.

Harrington, E. A., Bennett, M. R., Fanidi, A., and Evan, G. I. (1994) c-Myc-induced apoptosis in fibroblasts is inhibited by specific cytokines. *EMBO J.,* **13,** 3286–3295.

Hartley, A., Stone, J. M., Heron, C., Cooper, J. M., and Schapira, A. H. (1994) Complex I inhibitors induce dose-dependent apoptosis in PC12 cells: Relevance to Parkinson's disease. *J. Neurochem.,* **63,** 1987–1990.

Hashimoto, N., Feener, E. P., Zhang, W. R., and Goldstein, B. J. (1992) Insulin receptor protein-tyrosine phosphatases. Leukocyte common antigen-related phosphatase rapidly deactivates the insulin receptor kinase by preferential dephosphorylation of the receptor regulatory domain. *J. Biol. Chem.,* **267,** 13811–13814.

Heck, S., Lezoualc'h, F., Engert, S., and Behl, C. (1999) Insulin-like growth factor-1-mediated neuroprotection against oxidative stress is associated with activation of nuclear factor kappaB. *J. Biol. Chem.,* **274,** 9828–9835.

Helkala, E. L., Niskanen, L., Viinamaki, H., Partanen, J., and Uusitupa, M. (1995) Short-term and long-term memory in elderly patients with NIDDM. *Diabetes Care,* **18,** 681–685.

Heron-Milhavet, L., Karas, M., Goldsmith, C. M., Baum, B. J., and LeRoith, D. (2001) Insulin-like growth factor-I (IGF-I) receptor activation rescues UV-damaged cells through a p38 signaling pathway. Potential role of the IGF-I receptor in DNA repair. *J. Biol. Chem.,* **276,** 18185–18192.

Hetts, S. W., Rosen, K. M., Dikkes, P., Villa-Komaroff, L., and Mozell, R. L. (1997) Expression and imprinting of the insulin-like growth factor II gene in neonatal mouse cerebellum. *J. Neurosci. Res.,* **50,** 958–966.

Hill, P. A., Tumber, A., and Meikle, M. C. (1997) Multiple extracellular signals promote osteoblast survival and apoptosis. *Endocrinology,* **138,** 3849–3858.

Hong, F., Kwon, S. J., Jhun, B. S., Kim, S. S., Ha, J., Kim, S. J., Sohn, N. W., Kang, C., and Kang, I. (2001) Insulin-like growth factor-1 protects H9c2 cardiac myoblasts from oxidative stress-induced apoptosis via phosphatidylinositol 3-kinase and extracellular signal-regulated kinase pathways. *Life Sci.,* **68,** 1095–1105.

Iida, K. T., Suzuki, H., Sone, H., Shimano, H., Toyoshima, H., Yatoh, S., Asano, T., Okuda, Y., and Yamada, N. (2002) Insulin inhibits apoptosis of macrophage cell line, THP-1 cells, via phosphatidylinositol-3-kinase-dependent pathway. *Arterioscle. Thromb. Vasc. Biol.,* **22,** 380–386.

Ishii, D. N. (1995) Implication of insulin-like growth factors in the pathogenesis of diabetic neuropathy. *Brain Res. Brain Res. Rev.,* **20,** 47–67.

Ishii, D. N., and Lupien, S. B. (1995) Insulin-like growth factors protect against diabetic neuropathy: Effects on sensory nerve regeneration in rats. *J. Neurosci. Res.,* **40,** 138–144.

Ishii, D. N., Recio-Pinto, E., Spinelli, W., Mill, J. F., and Sonnenfeld, K. H. (1985) Neurite formation modulated by nerve growth factor, insulin, and tumor promoter receptors. *Int. J. Neurosci.,* **26,** 109–127.

Jakobsen, J., Sidenius, P., Gundersen, H. J., and Østerby, R. (1987) Quantitative changes of cerebral neocortical structure in insulin-treated long-term streptozocin-induced diabetes in rats. *Diabetes,* **36,** 597–601.

Janssen, J. A., and Lamberts, S. W. (2000) Circulating IGF-I and its protective role in the pathogenesis of diabetic angiopathy. *Clin. Endocrinol. (Oxf).,* **52,** 1–9.

Janssen, J. A., and Lamberts, S. W. (2002) The role of IGF-I in the development of cardiovascular disease in type 2 diabetes mellitus: Is prevention possible? *Eur. J. Endocrinol.,* **146,** 467–477.

Jiang, F., Frederick, T. J., and Wood, T. L. (2001) IGF-I synergizes with FGF-2 to stimulate oligodendrocyte progenitor entry into the cell cycle. *Dev. Biol.,* **232,** 414–423.

Jones, J. I., D'Ercole, A. J., Camacho-Hubner, C., Clemmons, D. R. (1991) Phosphorylation of insulin-like growth factor (IGF)-binding protein 1 in cell culture and in vivo: Effects on affinity for IGF-I. *Proc. Natl. Acad. Sci. U. S. A.,* **88,** 7481–7485.

Jones, J. I., Gockerman, A., Busby, W. H., Jr., Camacho-Hubner, C., and Clemmons, D. R. (1993) Extracellular matrix contains insulin-like growth factor binding protein-5: Potentiation of the effects of IGF-I. *J. Cell Biol.,* **121,** 679–687.

Kamal, A., Biessels, G. J., Duis, S. E., and Gispen, W. H. (2000) Learning and hippocampal synaptic plasticity in streptozotocin-diabetic rats: Interaction of diabetes and ageing. *Diabetologia,* **43,** 500–506.

Kamijo, M., Cherian, P. V., and Sima, A. A. F. (1993) The preventive effect of aldose reductase inhibition on diabetic optic neuropathy in the BB/W-rat. *Diabetologia,* **36,** 893–898.

Kanje, M., Skottner, A., Sjöberg, J., and Lundborg, G. (1989) Insulin-like growth factor-I (IGF-I) stimulates regeneration of the rat sciatic nerve. *Brain Res.,* **486,** 396–398.

Kanter-Lewensohn, L., Dricu, A., Girnita, L., Wejde, J., and Larsson, O. (2000) Expression of insulin-like growth factor-1 receptor (IGF-IR) and p27Kip1 in melanocytic tumors: A potential regulatory role of IGF-1 pathway in distribution of p27Kip1 between different cyclins. *Growth Factors,* **17,** 193–202.

Katz, J., Weiss, H., Goldman, B., Kanety, H., Stannard, B., LeRoith, D., and Shemer, J. (1995) Cytokines and growth factors modulate cell growth and insulin-like growth factor binding protein secretion by the human salivary cell line (HSG). *J. Cell. Physiol.,* **165,** 223–227.

Katz, L. E., Satin-Smith, M. S., Collett-Solberg, P., Baker, L., Stanley, C. A., and Cohen, P. (1998) Dual regulation of insulin-like growth factor binding protein-1 levels by insulin and cortisol during fasting. *J. Clin. Endocrinol. Metab.,* **83,** 4426–4430.

Kawakami, A., Nakashima, T., Tsuboi, M., Urayama, S., Matsuoka, N., Ida, H., Kawabe, Y., Sakai, H., Migita, K., Aoyagi, T., Nakashima, M., Maeda, K., and Eguchi, K. (1998) Insulin-like growth factor I stimulates proliferation and Fas-mediated apoptosis of human osteoblasts. *Biochem. Biophys. Res. Commun.,* **247,** 46–51.

Kelley, K. W., Meier, W. A., Minshall, C., Schacher, D. H., Liu, Q., VanHoy, R., Burgess, W., and Dantzer, R. (1998) Insulin growth factor-I inhibits apoptosis in hematopoietic progenitor cells. Implications in thymic aging. *Ann. N.Y. Acad. Sci.,* **840,** 518–524.

Kermer, P., Klocker, N., Labes, M., and Bahr, M. (2000) Insulin-like growth factor-I protects axotomized rat retinal ganglion cells from secondary death via PI3-K-dependent Akt phosphorylation and inhibition of caspase-3 in vivo. *J. Neurosci.,* **20,** 2–8.

Kiess, W., Yang, Y., Kessler, U., and Hoeflich, A. (1994) Insulin-like growth factor II (IGF-II) and the IGF-II/mannose-6-phosphate receptor: The myth continues. *Horm. Res.,* **41(Suppl 2),** 66–73.

Kihara, S., Shiraishi, T., Nakagawa, S., Toda, K., and Tabuchi, K. (1994) Visualization of DNA double strand breaks in the gerbil hippocampal CA1 following transient ischemia. *Neurosci. Lett.,* **175,** 133–136.

Kim, B., Leventhal, P. S., Saltiel, A. R., and Feldman, E. L. (1997) Insulin-like growth factor-I-mediated neurite outgrowth in vitro requires mitogen-activated protein kinase activation. *J. Biol. Chem.,* **272,** 21268–21273.

Kjeldsen, T., Andersen, A. S., Wiberg, F. C., Rasmussen, J. S., Schaffer, L., Balschmidt, P., Moller, K. B., and Moller, N. P. (1991) The ligand specificities of the insulin receptor and the insulin-like growth factor I receptor reside in different regions of a common binding site. *Proc. Natl. Acad. Sci. U. S. A.,* **88,** 4404–4408.

Kohner, E. M. (1987) Pathogenetic mechanisms in the development of diabetic retinopathy. In: *Diabetic Complications. Early Diagnosis and Treatment,* Edited by Andreani, D., Crepaldi, G., DiMario, U., and Pozza, G., pp. 55–64. Chichester, UK, John Wiley and Sons.

Kops, G. J., and Burgering, B. M. (2000) Forkhead transcription factors are targets of signalling by the proto-oncogene PKB (C-AKT). *J. Anat.,* **197(Pt 4),** 571–574.

Kramer, L., Fasching, P., Madl, C., Schneider, B., Damjancic, P., Waldhausl, W., Irsigler, K., and Grimm, G. (1998) Previous episodes of hypoglycemic coma are not associated with permanent cognitive brain dysfunction in IDDM patients on intensive insulin treatment. *Diabetes,* **47,** 1909–1914.

Kulik, G., Klippel, A., and Weber, M. J. (1997) Antiapoptotic signalling by the insulin-like growth factor I receptor, phosphatidylinositol 3-kinase, and Akt. *Mol. Cell. Biol.,* **17,** 1595–1606.

Kummer, J. L., Rao, P. K., and Heidenreich, K. A. (1997) Apoptosis induced by withdrawal of trophic factors is mediated by p38 mitogen-activated protein kinase. *J. Biol. Chem.,* **272,** 20490–20494.

Lackovic, Z., Salkovic, M., Kuci, Z., and Relja, M. (1990) Effect of long-lasting diabetes mellitus on rat and human brain monoamines. *J. Neurochem.,* **54,** 143–147.

Landau, D., Chin, E., Bondy, C., Domene, H., Roberts, C. T., Jr., Gronbaek, H., Flyvbjerg, A., and LeRoith, D. (1995) Expression of insulin-like growth factor binding proteins in the rat kidney: Effects of long-term diabetes. *Endocrinology,* **136,** 1835–1842.

Le Roith, D. (1999) Insulin-like growth factor. *Horm. Metab. Res.,* **31,** 41–42.

Lee-Kwon, W., Park, D., Baskar, P. V., Kole, S., and Bernier, M. (1998) Antiapoptotic signaling by the insulin receptor in Chinese hamster ovary cells. *Biochemistry,* **37,** 15747–15757.

Leverrier, Y., Thomas, J., Mathieu, A. L., Low, W., Blanquier, B., and Marvel, J. (1999) Role of PI3-kinase in Bcl-X induction and apoptosis inhibition mediated by IL-3 or IGF-1 in Baf-3 cells. *Cell Death Differ.,* **6,** 290–296.

Li, S., Ferber, A., Miura, M., and Baserga, R. (1994) Mitogenicity and transforming activity of the insulin-like growth factor-I receptor with mutations in the tyrosine kinase domain. *J. Biol. Chem.,* **269,** 32558–32564.

Li, Y., Sharov, V. G., Jiang, N., Zaloga, C., Sabbah, H. N., and Chopp, M. (1995) Ultrastructural and light microscopic evidence of apoptosis after middle cerebral artery occlusion in the rat. *Am. J. Pathol.,* **146,** 1045–1051.

Li, Z. G., Zhang, W., Grunberger, G., and Sima, A. A. F. (2002a) Hippocampal neuronal apoptosis in type 1 diabetes. *Brain Res.,* **946,** 221–231.

Li, Z. G., Zhang, W., and Sima, A. A. F. (2001) The effect of C-peptide on cell proliferation and apoptosis in human neuroblastoma cells. *Diabetologia,* **44(Suppl 1),** A297.

Li, Z. G., Zhang, W., and Sima, A. A. F. (2002b) C-peptide prevents hippocampal apoptosis in type 1 diabetes. *Int. J. Exp. Diabetes Res.,* **3,** 241–245.

Linnik, M. D., Zobrist, R. H., and Hatfield, M. D. (1993) Evidence supporting a role for programmed cell death in focal cerebral ischemia in rats. *Stroke,* **24,** 2002–2008; discussion 2008–2009.

Liu, Q., Schacher, D., Hurth, C., Freund, G. G., Dantzer, R., and Kelley, K. W. (1997) Activation of phosphatidylinositol 3′-kinase by insulin-like growth factor-I rescues promyeloid cells from apoptosis and permits their differentiation into granulocytes. *J. Immunol.,* **159,** 829–837.

Loo, D. T., Copani, A., Pike, C. J., Whittemore, E. R., Walencewicz, A. J., and Cotman, C. W. (1993) Apoptosis is induced by beta-amyloid in cultured central nervous system neurons. *Proc. Natl. Acad. Sci. U. S. A.,* **90,** 7951–7955.

Luo, J. M., and Murphy, L. J. (1991) Differential expression of insulin-like growth factor-I and insulin-like growth factor binding protein-1 in the diabetic rat. *Mol. Cell. Biochem.,* **103,** 41–50.

Luse, S. A. (1970) The ultrastructure of the brain in the diabetic Chinese hamster with special reference to synaptic abnormalities. *Electroencephalogr. Clin. Neurophysiol.,* **29,** 410.

Maegawa, H., McClain, D. A., Freidenberg, G., Olefsky, J. M., Napier, M., Lipari, T., Dull, T. J., Lee, J., and Ullrich, A. (1988) Properties of a human insulin receptor with a COOH-terminal truncation. II. Truncated receptors have normal kinase activity but are defective in signaling metabolic effects. *J. Biol. Chem.,* **263,** 8912–8917.

Marshall, S. M., and Alberti, K. G. (1989) Comparison of the prevalence and associated features of abnormal albumin excretion in insulin-dependent and non-insulin-dependent diabetes. *Q. J. Med.,* **70,** 61–71.

Matthews, C. C., and Feldman, E. L. (1996) Insulin-like growth factor I rescues SH-SY5Y human neuroblastoma cells from hyperosmotic induced programmed cell death. *J. Cell. Physiol.,* **166,** 323–331.

Mattson, M. P. (2000) Apoptosis in neurodegenerative disorders. *Nat. Rev. Mol. Cell. Biol.,* **1,** 120–129.

McCall, A. L. (1992) The impact of diabetes on the CNS. *Diabetes,* **41,** 557–570.

McMorris, F. A., and McKinnon, R. D. (1996) Regulation of oligodendrocyte development and CNS myelination by growth factors: Prospects for therapy of demyelinating disease. *Brain Pathol.,* **6,** 313–329.

Migdalis, I. N., Kalogeropoulou, K., Kalantzis, L., Nounopoulos, C., Bouloukos, A., and Samartzis, M. (1995) Insulin-like growth factor-I and IGF-I receptors in diabetic patients with neuropathy. *Diabet. Med.,* **12,** 823–827.

Miura, M., Li, S., and Baserga, R. (1995a) Effect of a mutation at tyrosine 950 of the insulin-like growth factor I receptor on the growth and transformation of cells. *Cancer Res.,* **55,** 663–667.

Miura, M., Surmacz, E., Burgaud, J. L., and Baserga, R. (1995b) Different effects on mitogenesis and transformation of a mutation at tyrosine 1251 of the insulin-like growth factor I receptor. *J. Biol. Chem.,* **270,** 22639–22644.

Mizuno, Y., Mochizuki, H., Sugita, Y., and Goto, K. (1998) Apoptosis in neurodegenerative disorders. *Intern. Med.,* **37,** 192–193.

Mogensen, C. E. (1998) *The Kidney and Hypertension in Diabetes Mellitus.* Boston, Kluwer Academic Publishers.

Mooradian, A. D. (1997) Central nervous system complications of diabetes mellitus—a perspective from the blood-brain barrier. *Brain Res. Brain Res. Rev.,* **23,** 210–218.

Mooradian, A. D., Perryman, K., Fitten, J., Kavonian, G. D., and Morley, J. E. (1988) Cortical function in elderly non-insulin dependent diabetic patients. Behavioral and electrophysiologic studies. *Arch. Intern. Med.,* **148,** 2369–2372.

Moorehead, R. A., Fata, J. E., Johnson, M. B., and Khokha, R. (2001) Inhibition of mammary epithelial apoptosis and sustained phosphorylation of Akt/PKB in MMTV-IGF-II transgenic mice. *Cell Death Differ.,* **8,** 16–29.

Morrione, A., Romano, G., Navarro, M., Reiss, K., Valentinis, B., Dews, M., Eves, E., Rosner, M. R., and Baserga, R. (2000) Insulin-like growth factor I receptor signaling in differentiation of neuronal H19-7 cells. *Cancer Res.,* **60,** 2263–2272.

Mozell, R. L., and McMorris, F. A. (1991) Insulin-like growth factor I stimulates oligodendrocyte development and myelination in rat brain aggregate cultures. *J. Neurosci. Res.,* **30,** 382–390.

Murakawa, Y., Zhang, W., Pierson, C. R., Brismar, T., Ostenson, C. G., Efendic, S., and Sima, A. A. F. (2002) Impaired glucose tolerance and insulinopenia in the GK-rat causes peripheral neuropathy. *Diabetes Metab. Res. Rev.,* **18,** 473–483.

Myers, M. G., Backer, J. M., Siddle, K., and White, M. F. (1991) The insulin receptor functions normally in Chinese hamster ovary cells after truncation of the C terminus. *J. Biol. Chem.,* **266,** 10616–10623.

Navarro, P., Valverde, A. M., Rohn, J. L., Benito, M., and Lorenzo, M. (2000) Akt mediates insulin rescue from apoptosis in brown adipocytes: Effect of ceramide. *Growth Horm. IGF Res.,* **10,** 256–266.

Nitatori, T., Sato, N., Waguri, S., Karasawa, Y., Araki, H., Shibanai, K., Kominami, E., and Uchiyama, Y. (1995) Delayed neuronal death in the CA1 pyramidal cell layer of the gerbil hippocampus following transient ischemia is apoptosis. *J. Neurosci.,* **15,** 1001–1011.

Normann, E. K., Evald, U., Dahl-Jörgensen, K., Larsen, S., Norman, N., and Tuvemo, T. (1994) Decreased serum insulin-like growth factor I during puberty in children with insulin dependent diabetes mellitus (IDDM). *Ups. J. Med. Sci.,* **99,** 147–154.

Ochoa, A., Domenzain, C., Clapp, C., and Martinez de la Escalera, G. (1997) Differential effects of basic fibroblast growth factor, epidermal growth factor, transforming growth factor-alpha, and insulin-like growth factor-I on a hypothalamic gonadotropin-releasing hormone neuronal cell line. *J. Neurosci. Res.,* **49,** 739–749.

O'Connor, R., Kauffmann-Zeh, A., Liu, Y., Lehar, S., Evan, G. I., Baserga, R., and Blatter, W. A. (1997) Identification of domains of the insulin-like growth factor I receptor that are required for protection from apoptosis. *Mol. Cell. Biol.,* **17,** 427–435.

Offen, D., Hochman, A., Gorodin, S., Ziv, I., Shirvan, A., Barzilai, A., and Melamed, E. (1999) Oxidative stress and neuroprotection in Parkinson's disease: Implications from studies on dopamine-induced apoptosis. *Adv. Neurol.,* **80,** 265–269.

Okubo, Y., Blakesley, V. A., Stannard, B., Gutkind, S., and Le Roith, D. (1998) Insulin-like growth factor-I inhibits the stress-activated protein kinase/c-Jun N-terminal kinase. *J. Biol. Chem.,* **273,** 25961–25966.

Pantoni, L., and Garcia, J. H. (1997) Pathogenesis of leukoariosis: A review. *Stroke,* **28,** 652–659.

Park, I. S., Kiyomoto, H., Alvarez, F., Xu, Y. C., Abboud, H. E., and Abboud, S. L. (1998) Preferential expression of insulin-like growth factor binding proteins-1, -3, and -5 during early diabetic renal hypertrophy in rats. *Am. J. Kidney Dis.,* **32,** 1000–1010.

Parrizas, M., Blakesley, V. A., Beitner-Johnson, D., and Le Roith, D. (1997a) The proto-oncogene Crk-II enhances apoptosis by a Ras-dependent, Raf-1/MAP kinase-independent pathway. *Biochem. Biophys. Res. Commun.,* **234,** 616–620.

Parrizas, M., Saltiel, A. R., and LeRoith, D. (1997b) Insulin-like growth factor 1 inhibits apoptosis using the phosphatidylinositol 3′-kinase and mitogen-activated protein kinase pathways. *J. Biol. Chem.,* **272,** 154–161.

Perks, C. M., McCaig, C., and Holly, J. M. (2000) Differential insulin-like growth factor (IGF)-independent interactions of IGF binding protein-3 and IGF binding protein-5 on apoptosis in human breast cancer cells. Involvement of the mitochondria. *J. Cell. Biochem.,* **80,** 248–258.

Pierson, C. R., Murakawa, Y., Zhang, W., and Sima, A. A. F. (2002a) Immediate early gene responses and nerve fiber regeneration differ between type 1 and type 2 neuropathies. *Diabetes,* **51(Suppl 2),** A200.

Pierson, C. R., Zhang, W., Murakawa, Y., and Sima, A. A. F. (2002b) Early gene responses of trophic factors in nerve regeneration differ in experimental type 1 and type 2 diabetic polyneuropathies. *J. Neuropathol. Exp. Neurol.,* **61,** 857–871.

Pierson, C. R., Zhang, W., and Sima, A. A. F. (2003) Proinsulin C-peptide replacement in type 1 diabetic BB/Wor-rats prevents deficits in nerve fiber regeneration. *J. Neuropathol. Exp. Neurol.,* **62,** 765–779.

Pirart, J. (1977) [Diabetes mellitus and its degenerative complications: A prospective study of 4,400 patients observed between 1947 and 1973 (author's translation)]. *Diabetes Metab.,* **3,** 97–107.

Popkin, M. K. (1997) Neurobehavioral aspects of diabetes mellitus: An overview. *Semin. Clin. Neuropsychiatry,* **2,** 2–3.

Popkin, M. K., Callies, A. L., Lentz, R. D., Colon, E. A., and Sutherland, D. E. (1988) Prevalence of major depression, simple phobia, and other psychiatric disorders in patients with long-standing type I diabetes mellitus. *Arch. Gen. Psychiatry,* **45,** 64–68.

Popovic, M., Biessels, G. J., Isaacson, R. L., and Gispen, W. H. (2001) Learning and memory in streptozotocin-induced diabetic rats in a novel spatial/object discrimination task. *Behav. Brain Res.,* **122,** 201–207.

Pozzessere, G., Rizzo, P. A., Valle, E., Mollica, M. A., Meccia, A., Morano, S., Di Mario, U., Andreani, D., and Morocutti, C. (1988) Early detection of neurological involvement in IDDM and NIDDM. Multimodal evoked potentials versus metabolic control. *Diabetes Care,* **11,** 473–480.

Pozzessere, G., Valle, E., de Crignis, S., Cordischi, V. M., Fattapposta, F., Rizzo, P. A., Pietravalle, P., Cristina, G., Morano, S., and di Mario, U. (1991) Abnormalities of cognitive functions in IDDM revealed by P300 event-related potential analysis. Comparison with short-latency evoked potentials and psychometric tests. *Diabetes,* **40,** 952–958.

Price, G. J., Berka, J. L., Werther, G. A., and Bach, L. A. (1997) Cell-specific regulation of mRNAs for IGF-I and IGF-binding proteins-4 and -5 in streptozotocin-diabetic rat kidney. *J. Mol. Endocrinol.,* **18,** 5–14.

Pu, S. F., Zhuang, H. X., Marsh, D. J., and Ishii, D. N. (1999) Insulin-like growth factor-II increases and IGF is required for postnatal rat spinal motoneuron survival following sciatic nerve axotomy. *J. Neurosci. Res.,* **55,** 9–16.

Pugazhenthi, S., Boras, T., O'Connor, D., Meintzer, M. K., Heidenreich, K. A., and Reusch, J. E. (1999) Insulin-like growth factor I-mediated activation of the transcription factor cAMP response element-binding protein in PC12 cells. Involvement of p38 mitogen-activated protein kinase-mediated pathway. *J. Biol. Chem.,* **274,** 2829–2837.

Qian, H., Hausman, D. B., Compton, M. M., Martin, R. J., Della-Fera, M. A., Hartzell, D. L., and Baile, C. A. (2001) TNFalpha induces and insulin inhibits caspase 3-dependent adipocyte apoptosis. *Biochem. Biophys. Res. Commun.,* **284,** 1176–1183.

Raev, D. C. (1993) Evolution of cardiac changes in young insulin-dependent (type 1) diabetic patients—one more piece of the puzzle of diabetic cardiopathy. *Clin. Cardiol.,* **16,** 784–790.

Recio-Pinto, E., and Ishii, D. N. (1984) Effects of insulin, insulin-like growth factor-II and nerve growth factor on neurite outgrowth in cultured human neuroblastoma cells. *Brain Res.,* **302,** 323–334.

Reske-Nielsen, E., Lundbaek, K., and O. J. R. (1965) Pathological changes in the central and peripheral nervous system of young long-term diabetics. *Diabetologia,* **1,** 233–241.

Riaz, S. S., and Tomlinson, D. R. (1996) Neurotrophic factors in peripheral neuropathies: Pharmacological strategies. *Prog. Neurobiol.,* **49,** 125–143.

Rieu, M., and Binoux, M. (1985) Serum levels of insulin-like growth factor (IGF) and IGF binding protein in insulin-dependent diabetics during an episode of severe metabolic decompensation and the recovery phase. *J. Clin. Endocrinol. Metab.,* **60,** 781–785.

Rind, H. B., and von Bartheld, C. S. (2002) Target-derived cardiotrophin-1 and insulin-like growth factor-I promote neurite growth and survival of developing oculomotor neurons. *Mol. Cell. Neurosci.,* **19,** 58–71.

Rinderknecht, E., and Humbel, R. E. (1978) The amino acid sequence of human insulin-like growth factor I and its structural homology with proinsulin. *J. Biol. Chem.,* **253,** 2769–2776.

Ristic, H., Srinivasan, S., Hall, K. E., Sima, A. A. F., and Wiley, J. W. (1998) Serum from diabetic BB/W rats enhances calcium currents in primary sensory neurons. *J. Neurophysiol.,* **80,** 1236–1244.

Rodgers, B. D., Bautista, R. M., and Nicoll, C. S. (1995) Regulation of insulin-like growth factor-binding proteins in rats with insulin-dependent diabetes mellitus. *Proc. Soc. Exp. Biol. Med.,* **210,** 234–241.

Russell, J. W., Cheng, H. L., and Golovoy, D. (2000) Insulin-like growth factor-I promotes myelination of peripheral sensory axons. *J. Neuropathol. Exp. Neurol.,* **59,** 575–584.

Russell, J. W., Sullivan, K. A., Windebank, A. J., Herrmann, D. N., and Feldman, E. L. (1999) Neurons undergo apoptosis in animal and cell culture models of diabetes. *Neurobiol. Dis.,* **6,** 347–363.

Ryu, B. R., Ko, H. W., Jou, I., Noh, J. S., and Gwag, B. J. (1999) Phosphatidylinostiol 3-kinase-mediated regulation of neuronal apoptosis and necrosis by insulin and IGF-I. *J. Neurobiol.,* **39,** 536–546.

Saeki, M., Maeda, S., Wada, K., and Kamisaki, Y. (2002) Insulin-like growth factor-1 protects peroxynitrite-induced cell death by preventing cytochrome c-induced caspase-3 activation. *J. Cell. Biochem.,* **84,** 708–716.

Salkovic, M., Sabolic, I., and Lackovic, Z. (1995) Striatal dopaminergic D1 and D2 receptors after intracerebroventricular application of alloxan and streptozocin in rat. *J. Neural Transm. Gen. Sect.,* **100,** 137–145.

Sara, V. R., Carlsson-Skwirut, C., Bergman, T., Jörnvall, H., Roberts, P. J., Crawford, M., Håkansson, L. N., Civalero, I., and Nordberg, A. (1989) Identification of Gly-Pro-Glu (GPE), the aminoterminal tripeptide of insulin-like growth factor 1 which is truncated in brain, as a novel neuroactive peptide. *Biochem. Biophys. Res. Commun.,* **165,** 766–771.

Scheid, M. P., Schubert, K. M., and Duronio, V. (1999) Regulation of bad phosphorylation and association with Bcl-x(L) by the MAPK/Erk kinase. *J. Biol. Chem.,* **274,** 31108–31113.

Schoenle, E. J., Schoenle, D., Molinari, L., and Largo, R. H. (2002) Impaired intellectual development in children with type 1 diabetes: Association with HbA1c, age at diagnosis and sex. *Diabetologia,* **45,** 108–114.

Selinfreund, R. H., and Blair, L. A. (1994) Insulin-like growth factor-I induces a rapid increase in calcium currents and spontaneous membrane activity in clonal pituitary cells. *Mol. Pharmacol.,* **45,** 1215–1220.

Sell, C., Baserga, R., and Rubin, R. (1995) Insulin-like growth factor I (IGF-I) and the IGF-I receptor prevent etoposide-induced apoptosis. *Cancer Res.,* **55,** 303–306.

Sima, A. A. F. (1996) Metabolic alterations of peripheral nerve in diabetes. *Semin. Neurol.,* **16,** 129–137.

Sima, A. A. F. (1997) Pathological definition and evaluation of diabetic neuropathy and clinical correlations. *Can. J. Neurol. Sci.,* **21(Suppl 4),** 513–517.

Sima, A. A. F., Lattimer, S. A., Yagihashi, S., and Greene, D. A. (1986) Axo-glial dysjunction. A novel structural lesion that accounts for poorly reversible slowing of nerve conduction in the spontaneously diabetic bio-breeding rat. *J. Clin. Invest.,* **77,** 474–484.

Sima, A. A. F., Merry, A. C., Hall, D. E., Grant, M., Murray, F. T., and Guberski, D. (1997a) The BB/ZDR-rat; A model for type II diabetic neuropathy. *Exp. Clin. Endocrinol. Diabetes,* **105,** 62–63.

Sima, A. A. F., Merry, A. C., and Levitan, I. (1997b) Increased regeneration in ARI-treated diabetic nerve is associated with upregulation of IGF-1 and NGF receptors. *Exp. Clin. Endocrinol. Diabetes,* **105,** 60–62.

Sima, A. A. F., Murakawa, Y., Zhang, W., Wahren, J., and Pierson, C. R. (2001a) Insulin receptor isoform expression in sciatic nerve and dorsal root ganglia in type-1 BB/W-, BB/W C-peptide treated and type-2 BB/Z-diabetic rats. *Int. J. Exp. Diabetes Res.,* **2,** 288.

Sima, A. A. F., Nathaniel, V., Bril, V., McEwen, T. A., and Greene, D. A. (1988) Histopathological heterogeneity of neuropathy in insulin-dependent and non-insulin-dependent diabetes, and demonstration of axo-glial dysjunction in human diabetic neuropathy. *J. Clin. Invest.,* **81,** 349–364.

Sima, A. A. F., and Sugimoto, K. (1999) Experimental diabetic neuropathy: An update. *Diabetologia,* **42,** 773–788.

Sima, A. A. F., Thomas, P. K., Ishii, D., and Vinik, A. (1997c) Diabetic neuropathies. *Diabetologia,* **40(Suppl 3),** B74–B77.

Sima, A. A. F., Zhang, W., Cherian, P. V., and Chakrabarti, S. (1992) Impaired visual evoked potential and primary axonopathy of the optic nerve in the diabetic BB/W-rat. *Diabetologia,* **35,** 602–607.

Sima, A. A. F., Zhang, W., Murakawa, Y., Wahren, J., and Pierson, C. R. (2002) C-peptide corrects aberrations in immediate early gene responses and cytoskeletal protein expression in regenerating nerve in type 1 neuropathy. *Diabetes,* **51(Suppl 2),** A198.

Sima, A. A. F., Zhang, W., Sugimoto, K., Henry, D., Li, Z., Wahren, J., and Grunberger, G. (2001b) C-peptide prevents and improves chronic Type I diabetic polyneuropathy in the BB/Wor rat. *Diabetologia,* **44,** 889–897.

Sima, A. A. F., Zhang, W., Xu, G., Sugimoto, K., Guberski, D., and Yorek, M. A. (2000) A comparison of diabetic polyneuropathy in type II diabetic BBZDR/Wor rats and in type I diabetic BB/Wor rats. *Diabetologia,* **43,** 786–793.

Singleton, J. R., Randolph, A. E., and Feldman, E. L. (1996) Insulin-like growth factor I receptor prevents apoptosis and enhances neuroblastoma tumorigenesis. *Cancer Res.,* **56,** 4522–4529.

Sizonenko, S. V., Sirimanne, E. S., Williams, C. E., and Gluckman, P. D. (2001) Neuroprotective effects of the N-terminal tripetide of IGF-1, glycine-proline-glutamate, in the immature rat brain after hypoxic-ischemic injury. *Brain Res.,* **922,** 42–50.

Stefanis, L., Burke, R. E., and Greene, L. A. (1997) Apoptosis in neurodegenerative disorders. *Curr. Opin. Neurol.,* **10,** 299–305.

Stoll, G., and Muller, H. W. (1999) Nerve injury, axonal degeneration and neural regeneration: Basic insights. *Brain Pathol.,* **9,** 313–325.

Sugimoto, K., Murakawa, Y., and Sima, A. A. F. (2000a) Diabetic neuropathy—a continuing enigma. *Diabetes Metab. Res. Rev.,* **16,** 408–433.

Sugimoto, K., Murakawa, Y., and Sima, A. A. F. (2000b) Fine structural localization of insulin receptor in rat dorsal root ganglion and spinal cord. *Diabetes,* **49 (Suppl),** A168.

Sugimoto, K., Murakawa, Y., and Sima, A. A. F. (2002) Expression and localization of insulin receptor in rat dorsal root ganglion and spinal cord. *J. Peripher. Nerv. Syst.,* **7,** 44–53.

Sugimoto, K., Murakawa, Y., Zhang, W., Xu, G., and Sima, A. A. F. (2000c) Insulin receptor in rat peripheral nerve: Its localization and alternatively spliced isoforms. *Diabetes Metab. Res. Rev.,* **16,** 354–363.

Syroid, D. E., Zorick, T. S., Arbet-Engels, C., Kilpatrick, T. J., Eckhart, W., and Lemke, G. (1999) A role for insulin-like growth factor-I in the regulation of Schwann cell survival. *J. Neurosci.,* **19,** 2059–2068.

Takata, Y., Webster, N. J., and Olefsky, J. M. (1991) Mutation of the two carboxyl-terminal tyrosines results in an insulin receptor with normal metabolic signaling but enhanced mitogenic signaling properties. *J. Biol. Chem.,* **266,** 9135–9139.

Tan, K., and Baxter, R. C. (1986) Serum insulin-like growth factor I levels in adult diabetic patients: The effect of age. *J. Clin. Endocrinol. Metab.,* **63,** 651–655.

Tanaka, M., Sawada, M., Yoshida, S., Hanaoka, F., and Marunouchi, T. (1995) Insulin prevents apoptosis of external granular layer neurons in rat cerebellar slice cultures. *Neurosci. Lett.,* **199,** 37–40.

Tatton, N. A., Maclean-Fraser, A., Tatton, W. G., Perl, D. P., and Olanow, C. W. (1998) A fluorescent double-labeling method to detect and confirm apoptotic nuclei in Parkinson's disease. *Ann. Neurol.,* **44,** S142–S148.

Tatton, W. G., Chalmers-Redman, R. M., Ju, W. Y., Wadia, J., and Tatton, N. A. (1997) Apoptosis in neurodegenerative disorders: Potential for therapy by modifying gene transcription. *J. Neural Transm. Suppl.,* **49,** 245–268.

Tavare, J. M., and Denton, R. M. (1988) Studies on the autophosphorylation of the insulin receptor from human placenta. Analysis of the sites phosphorylated by two-dimensional peptide mapping. *Biochem. J.,* **252,** 607–615.

Terry, B., Cannon, J., Guong, L., Wands, J., and de la Monk, S. M. (2001) Abnormalities in insulin, IGF-I, and corresponding receptor (R) expression in Alzheimer disease. *J. Neuropathol. Exp. Neurol.,* **60,** 546.

Thomas, P. K. (1997) Classification, differential diagnosis, and staging of diabetic peripheral neuropathy. *Diabetes,* **46 (Suppl 2),** S54–S57.

Thrailkill, K. M. (2000) Insulin-like growth factor-I in diabetes mellitus: Its physiology, metabolic effects, and potential clinical utility. *Diabetes Technol. Ther.,* **2,** 69–80.

Tomlinson, D. R., and Fernyhough, P. (2000) Neurotrophism in diabetic neuropathy. In: *Chronic Complications in Diabetes. Frontiers in Animal Diabetes Research,* Edited by Sima, A. A. F., and Shafrir, E., pp. 167–182. Amsterdam: Harwood Academic Publishers.

Tornqvist, H. E., Pierce, M. W., Frackelton, A. R., Nemenoff, R. A., and Avruch, J. (1987) Identification of insulin receptor tyrosine residues autophosphorylated in vitro. *J. Biol. Chem.,* **262,** 10212–10219.

Tumber, A., Meikle, M. C., and Hill, P. A. (2000) Autocrine signals promote osteoblast survival in culture. *J. Endocrinol.,* **167,** 383–390.

Ullrich, A., Gray, A., Tam, A. W., Yang-Feng, T., Tsubokawa, M., Collins, C., Henzel, W., Le Bon, T., Kathuria, S., Chen, E., et al. (1986) Insulin-like growth factor I receptor primary structure: Comparison with insulin receptor suggests structural determinants that define functional specificity. *EMBO J.,* **5,** 2503–2512.

Ullrich, A., and Schlessinger, J. (1990) Signal transduction by receptors with tyrosine kinase activity. *Cell,* **61,** 203–212.

Valentinis, B., Morrione, A., Peruzzi, F., Prisco, M., Reiss, K., and Baserga, R. (1999) Anti-apoptotic signaling of the IGF-I receptor in fibroblasts following loss of matrix adhesion. *Oncogene,* **18,** 1827–1836.

van Golen, C. M., Castle, V. P., and Feldman, E. L. (2000) IGF-I receptor activation and BCL-2 overexpression prevent early apoptotic events in human neuroblastoma. *Cell Death Differ.,* **7,** 654–665.

van Golen, C. M., and Feldman, E. L. (2000) Insulin-like growth factor I is the key growth factor in serum that protects neuroblastoma cells from hyperosmotic-induced apoptosis. *J. Cell. Physiol.,* **182,** 24–32.

Vestling, M., Wiehager, B., Tanii, H., and Cowburn, R. F. (2001) Akt activity in presenilin 1 wild-type and mutation transfected human SH-SY5Y neuroblastoma cells after serum deprivation and high glucose stress. *J. Neurosci. Res.,* **66,** 448–456.

Vincent, A., and Feldman, E. (2002) Control of cell survival by IGF singnaling pathways. *Growth Horm. IGF Res.,* **12,** 193.

Vincent, A. M., Brownlee, M., and Russell, J. W. (2002) Oxidative stress and programmed cell death in diabetic neuropathy. *Ann. N.Y. Acad. Sci.,* **959,** 368–383.

Vinik, A. I., Holland, M. T., Le Beau, J. M., Liuzzi, F. J., Stansberry, K. B., and Colen, L. B. (1992) Diabetic neuropathies. *Diabetes Care,* **15,** 1926–1975.

Walter, H. J., Berry, M., Hill, D. J., Cwyfan-Hughes, S., Holly, J. M., and Logan, A. (1999) Distinct sites of insulin-like growth factor (IGF)-II expression and localization in lesioned rat brain: Possible roles of IGF binding proteins (IGFBPs) in the mediation of IGF-II activity. *Endocrinology,* **140,** 520–532.

Wang, C., Li, Y., Wible, B., Angelides, K. J., and Ishii, D. N. (1992) Effects of insulin and insulin-like growth factors on neurofilament mRNA and tubulin mRNA content in human neuroblastoma SH-SY5Y cells. *Brain Res. Mol. Brain Res.,* **13,** 289–300.

Weinberger, J., Biscarra, V., Weisberg, M. K., and Jacobson, J. H. (1983) Factors contributing to stroke in patients with atherosclerotic disease of the great vessels: The role of diabetes. *Stroke,* **14,** 709–712.

Welsh, B., and Wecker, L. (1991) Effects of streptozotocin-induced diabetes on acetylcholine metabolism in rat brain. *Neurochem. Res.,* **16,** 453–460.

White, M. F., Shoelson, S. E., Keutmann, H., and Kahn, C. R. (1988) A cascade of tyrosine autophosphorylation in the beta-subunit activates the phosphotransferase of the insulin receptor. *J. Biol. Chem.,* **263,** 2969–2980.

Wilkins, H. R., Ohneda, K., Keku, T. O., D'Ercole, A. J., Fuller, C. R., Williams, K. L., and Lund, P. K. (2002) Reduction of spontaneous and irradiation-induced apoptosis in small intestine of IGF-I transgenic mice. *Am. J. Physiol. Gastrointest. Liver Physiol.,* **283,** G457–G464.

Williams, C., Guan, J., Miller, O., Beilharz, E., McNeill, H., Sirimanne, E., and Gluckman, P. (1995) The role of the growth factors IGF-1 and TGF beta 1 after hypoxic-ischemic brain injury. *Ann. N.Y. Acad. Sci.,* **765,** 306–307.

Wuarin, L., Guertin, D. M., and Ishii, D. N. (1994) Early reduction in insulin-like growth factor gene expression in diabetic nerve. *Exp. Neurol.,* **130,** 106–114.

Wuarin, L., Namdev, R., Burns, J. G., Fei, Z. J., and Ishii, D. N. (1996) Brain insulin-like growth factor-II mRNA content is reduced in insulin-dependent and non-insulin-dependent diabetes mellitus. *J. Neurochem.,* **67,** 742–751.

Xu, G., Pierson, C. R., Murakawa, Y., and Sima, A. A. F. (2002) Altered tubulin and neurofilament expression and impaired axonal growth in diabetic nerve regeneration. *J. Neuropathol. Exp. Neurol.,* **61,** 164–175.

Xu, G., and Sima, A. A. F. (2001) Altered immediate early gene expression in injured diabetic nerve: Implications in regeneration. *J. Neuropathol. Exp. Neurol.,* **60,** 972–983.

Yagihashi, S. (1995) Pathology and pathogenetic mechanisms of diabetic neuropathy. *Diabetes Metab. Rev.,* **11,** 193–225.

Yagihashi, S., Sugimoto, K., and Wada, R. (1994) Different neuropathic patterns between type-1 and type-2 animal models. In: *Pathogenesis and Treatment of NIDDM and its Related Problems,* Edited by Sakamoto, N., Alberti, K., and Hotta, N., pp. 401–405. Amsterdam, Elsevier.

Yamaguchi, A., Tamatani, M., Matsuzaki, H., Namikawa, K., Kiyama, H., Vitek, M. P., Mitsuda, N., and Tohyama, M. (2001) Akt activation protects hippocampal neurons from apoptosis by inhibiting transcriptional activity of p53. *J. Biol. Chem.,* **276,** 5256–5264.

Ye, P., Carson, J., and D'Ercole, A. J. (1995) In vivo actions of insulin-like growth factor-I (IGF-I) on brain myelination: Studies of IGF-I and IGF binding protein-1 (IGFBP-1) transgenic mice. *J. Neurosci.,* **15,** 7344–7356.

Zackenfels, K., Oppenheim, R. W., and Rohrer, H. (1995) Evidence for an important role of IGF-I and IGF-II for the early development of chick sympathetic neurons. *Neuron,* **14,** 731–741.

Zackenfels, K., and Rohrer, H. (1993) IGF-I stimulates chick sympathetic neuron proliferation in vitro and in vivo. *Ann. N.Y. Acad. Sci.,* **692,** 302–304.

Zawada, W. M., Zastrow, D. J., Clarkson, E. D., Adams, F. S., Bell, K. P., and Freed, C. R. (1998) Growth factors improve immediate survival of embryonic dopamine neurons after transplantation into rats. *Brain Res.,* **786,** 96–103.

Zhang, W., Ghetti, B., and Lee, W. H. (1997) Decreased IGF-I gene expression during the apoptosis of Purkinje cells in pcd mice. *Brain Res. Dev. Brain Res.,* **98,** 164–176.

Zhang, W., Li, Z. G., and Sima, A. A. F. (2001) The effect of C-peptide on cell proliferation of human neuroblastoma cell with and without Insulin or IGF-1. *Diabetes,* **50 (Suppl 2),** A190.

Zhang, W., Li, Z. G., and Sima, A. A. F. (2002) C-peptide potentiates the anti-apoptotic effect of insulin via activation of Bcl-2 and NF-kB. *Diabetes,* **51 (Suppl 2),** A197.

Zheng, W. H., Kar, S., and Quirion, R. (2002) Insulin-like growth factor-1-induced phosphorylation of transcription factor FKHRL1 is mediated by phosphatidylinositol 3-kinase/Akt kinase and role of this pathway in insulin-like growth factor-1-induced survival of cultured hippocampal neurons. *Mol. Pharmacol.,* **62,** 225–233.

Zhong, J., Deng, J., Ghetti, B., and Lee, W. H. (2002) Inhibition of insulin-like growth factor I activity contributes to the premature apoptosis of cerebellar granule neuron in weaver mutant mice: In vitro analysis. *J. Neurosci. Res.,* **70,** 36–45.

Zhuang, H. X., Wuarin, L., Fei, Z. J., and Ishii, D. N. (1997) Insulin-like growth factor (IGF) gene expression is reduced in neural tissues and liver from rats with non-insulin-dependent diabetes mellitus, and IGF treatment ameliorates diabetic neuropathy. *J. Pharmacol. Exp. Ther.,* **283,** 366–374.

Ziv, I., and Melamed, E. (1998) Role of apoptosis in the pathogenesis of Parkinson's disease: A novel therapeutic opportunity? *Mov. Disord.,* **13,** 865–870.

Zumkeller, W. (2002) The insulin-like growth factor system in hematopoietic cells. *Leukemia Lymphoma,* **43,** 487–491.

